# Combining Charlson comorbidity and VACS indices improves prognostic accuracy for all-cause mortality for patients with and without HIV in the Veterans Health Administration

**DOI:** 10.3389/fmed.2023.1342466

**Published:** 2024-01-31

**Authors:** Kathleen A. McGinnis, Amy C. Justice, Vincent C. Marconi, Maria C. Rodriguez-Barradas, Ronald G. Hauser, Krisann K. Oursler, Sheldon T. Brown, Kendall J. Bryant, Janet P. Tate, for the Veterans Aging Cohort Study

**Affiliations:** ^1^VA Connecticut Healthcare System, West Haven, CT, United States; ^2^Yale School of Medicine, New Haven, CT, United States; ^3^The Atlanta Veterans Affairs Medical Center, Emory University School of Medicine and Rollins School of Public Health, Atlanta, GA, United States; ^4^VA Medical Center, Decatur, GA, United States; ^5^Infectious Diseases Section, Michael E. DeBakey Veterans Affairs Medical Center, Houston, TX, United States; ^6^Department of Medicine, Baylor College of Medicine, Houston, TX, United States; ^7^Department of Laboratory Medicine, Yale University School of Medicine, New Haven, CT, United States; ^8^Department of Internal Medicine, Virginia Tech Carilion School of Medicine, Roanoke, VA, United States; ^9^VA Salem Healthcare System, Salem, VA, United States; ^10^James J. Peters VA Medical Center, Bronx, NY, United States; ^11^National Institute on Alcohol Abuse and Alcoholism, Bethesda, MD, United States

**Keywords:** VACS Index, Charlson Comorbidity Index, HIV, mortality, prediction

## Abstract

**Introduction:**

As people age with HIV (PWH), many comorbid diseases are more common than among age matched comparators without HIV (PWoH). While the Veterans Aging Cohort (VACS) Index 2.0 accurately predicts mortality in PWH using age and clinical biomarkers, the only included comorbidity is hepatitis C. We asked whether adding comorbid disease groupings from the Charlson Comorbidity Index (CCI) improves the accuracy of VACS Index.

**Methods:**

To maximize our ability to model mortality among older age groups, we began with PWoH in Veterans Health Administration (VA) from 2007–2017, divided into development and validation samples. Baseline predictors included age, and components of CCI and VACS Index (excluding CD4 count and HIV RNA). Patients were followed until December 31, 2021. We used Cox models to develop the VACS-CCI score and estimated mortality using a parametric (gamma) survival model. We compared accuracy using C-statistics and calibration curves in validation overall and within subgroups (gender, age </≥65 years, race/ethnicity, and CCI score). We then applied VACS-CCI in PWH and compared its accuracy to age, VACS Index 2.0, CCI and VACS-CCI with CD4 and HIV RNA added.

**Results:**

The analytic sample consisted of 6,588,688 PWoH and 30,539 PWH. Among PWoH/PWH, median age was 65/55 years; 6%/3% were women; 15%/48% were Black and 5%/7% Hispanic. VACS-CCI provided the best discrimination (C-statistic = 0.81) with excellent calibration (predicted and observed mortality largely overlapped) overall and within subgroups. When VACS-CCI was applied to PWH it demonstrated similar discrimination as VACS Index 2.0 (C-statistic = 0.77 for both) but superior calibration among those with CD4 < 200. Discrimination was improved when CD4 and HIV RNA were added VACS-CCI (C-statistic = 0.79). Liver and kidney disease, congestive heart failure, malignancy, and dementia were negatively associated with CD4 (*p*-trends all <0.0001).

**Discussion:**

Among PWH and PWoH in VA care, age alone weakly discriminates risk of mortality. VACS Index 2.0, CCI, and VACS-CCI all provide better discrimination, but VACS-CCI is more consistently calibrated. The association of comorbid diseases with lower CD4 underscores the likely role of HIV in non-AIDS conditions. Future work will include adding CD4 and HIV RNA to VACS-CCI and validating it in independent data.

## Introduction

With advances in antiretroviral treatment (ART), people with HIV (PWH) are typically able to achieve HIV RNA viral suppression and maintain high CD4 count. However, as PWH age, many non-HIV related comorbid diseases are more common than among age matched comparators without HIV (PWoH) (1–4), and disparities in life expectancy persist (5).

The observed shift from AIDS to non-AIDS morbidity prompted the development of the Veterans Aging Cohort Study (VACS) Index using data from the VACS-HIV cohort, which is comprised of Veterans Health Administration (VHA) electronic health record (EHR) data. The VACS Index incorporates general health (age, albumin, body mass index, hemoglobin, Fibrosis-4 Index for Liver Fibrosis [FIB-4], estimated glomerular filtration rate [eGFR], white blood count, hepatitis C virus [HCV]), and HIV-specific (HIV RNA and CD4 cell count) clinical data to characterize overall disease burden and reflect risk of mortality (6–8). Since its inception and validation, the VACS Index has consistently demonstrated its utility in discriminating mortality and other outcomes in various samples of PWH (7, 9–14). VACS Index risk estimates also generalize well to demographically similar people without HIV in VHA by assuming HIV RNA is undetectable and CD4 cell counts are normal (15). The VACS Index is available online at MDCalc^1^ (14). Our long-term goal is to provide automated, individualized estimates from these risk index tools in real time and at the point of care to facilitate informed medical decision making in people with and without HIV.

Although the biomarkers included in the VACS Index reflect physiologic injury from comorbid disease, the only comorbidity directly included is HCV. Comorbidities such as diabetes, pulmonary disease, cancer, cardiovascular disease, and others are not directly reflected in the biomarkers included in the VACS Index. The Charlson Comorbidity Index (CCI) estimates mortality risk based on common comorbid conditions and uses International Classification of Diseases (ICD) codes readily available in administrative billing data (16). The CCI is widely used in clinical research with over ten thousand citations in Pub Med but its discrimination of mortality is only fair (16).

We aimed to determine whether adding comorbid disease groupings from the CCI would improve the accuracy of the VACS Index in people with and without HIV in care in the VHA.

## Materials and methods

### Data

The VHA includes longitudinal, paperless, national EHR data on ~13.5 million Veterans who received care at over 1,200 points-of-care since 1999 and we capture this information in the VACS-National. To identify a reasonably current patient sample with adequate follow up, we restricted to patients with VHA healthcare visits 2007 through 2017.

#### Development and validation samples from the general VHA population

To maximize our ability to model mortality among older age groups, we began with the general VHA population that does not include PWH. For the development and validation samples, we excluded Veterans diagnosed with HIV at any time so that we could evaluate the VACS Index in a general, non-HIV sample. We used a two-stage sampling design to randomly select a single index visit per patient. In the first stage, we identified primary care or other clinic visits (using stop codes, Supplementary Table S1), that included a routine blood pressure reading. For each year from 2007 to 2017 we randomly selected one outpatient visit date per person that was at least 18 months after the date of the first outpatient diagnosis in the EHR, giving preference to a primary care visit if one was available. In the second stage, to ensure a range of follow-up times and disease severity, we pooled the visits from each year for those patients that had complete VACS Index data. Compared to those with complete data, patients who had visits with missing data were of similar age and sex, but more likely to have unknown race (8.9% vs. 5.7%), no comorbidities (58.5% vs. 46.2%), and a visit from 2007 to 2011 (48.1% vs. 41.0%). We then randomly selected a single visit per person from all eligible visits. This index visit date was used as the baseline for lab values, comorbidities and start of follow-up.

#### PWH validation sample from the VHA population

Using data from the VACS-HIV for the PWH sample, we used similar methods to the two-staged sampling design described above. However, we randomly selected one outpatient visit date per person that was at least 1 year after the date of the first ART prescription. HIV status was determined from ICD-10 codes as previously described (17).

#### Mortality data

Mortality data are from a combination of sources available in the VHA (Social Security Administration, Center for Medicare and Medicaid Services, VHA inpatient deaths and the VA Death Beneficiary database) with accuracy comparable to the National Death Index (18, 19). Patients were followed until death, 10 years, or 12/31/2021. This study was approved by Yale University and VA Connecticut Healthcare System institutional review boards.

#### Veterans Aging Cohort Study (VACS) Index

The development and internal validity of VACS Index has been described in detail elsewhere (7, 8). As in prior work, for the general non-HIV sample, we assumed CD4 cell count was normal (>500 CD4 cells/mm3) and HIV RNA was undetectable for people without HIV infection (7, 20, 21). In addition to these HIV specific laboratory tests, VACS Index 2.0 includes age, BMI and the following routinely monitored laboratory tests: hemoglobin, platelets, alanine and aspartate transaminases (ALT, AST), creatinine, white blood count, albumin, and HCV status.

Composite markers of liver and renal injury were computed. FIB-4, composed of AST, ALT, platelets, and age, is a validated indicator of liver fibrosis (22). Estimated glomerular filtration rate (eGFR; using the CKD-EPI 2021 equation, composed of serum creatinine, age, and gender, but not race) is a validated indicator of impaired renal function (23).

HCV status was defined as positive if the patient ever had a detectable virus, detectable HCV genotype, treatment for HCV, antibody positive (unless RNA negative) or ICD code for chronic HCV before the index date. Of those deemed HCV positive, 75% had detectable virus. We extracted values from the EHR, restricting to those obtained in outpatient settings, using those closest to the index visit date within 18 months before to 14 days after to allow for labs ordered at the visit. Because full liver panels are not always obtained, we set values to normal when albumin (4 g/dL), or one of ALT (25 IU/L) or AST (20 IU/L) was missing, resulting in complete data for 81% of otherwise eligible sample.

#### Charlson Comorbidity Index (CCI)

Components of the CCI (Table 1) were based on conditions documented by ICD-9 and ICD-10 diagnosis codes in the 2 years prior to index visit date. Conditions were considered present based on one or more inpatient or two or more outpatient codes. The ICD codes used to identify CCI comorbidities (Supplementary Table S2) were based on a careful examination of prior studies (24–27). The codes identified in these three sources substantially overlapped. A few codes differed due to Canadian versus US codes, errors, and omissions but the difference in CCI score using these sources was negligible.

**Table 1:** Characteristics of patients at randomly selected index visit, 2007–2017.

		People without HIV			People with HIV
		All	Development	Validation	
*N*		6,588,688	6,062,932	525,756	30,539
Year, No. (%)	2007–2011	2,615,193 (39.7)	2,615,193 (43.1)		11,192 (36.6)
	2012	525,756 (8.0)		525,756 (100.0)	2,370 (7.8)
	2013–2017	3,447,739 (52.3)	3,447,739 (56.9)		16,977 (55.6)
Deaths during maximum 10 years follow-up, No. (%)		2,286,881 (34.7)	2,082,928 (34.4)	203,953 (38.8)	9,005 (29.5)
Follow-up, years, Median (IQR)		6.3 (4.5–9.3)	6.2 (4.4–8.8)	9.4 (5.5–9.8)	6.5 (4.6–9.5)
Sex, No. (%)	Female	407,215 (6.2)	376,380 (6.2)	30,835 (5.9)	824 (2.7)
Race, No. (%)	White	4,497,943 (68.3)	4,134,920 (68.2)	363,023 (69.0)	11,522 (37.7)
	Black	979,166 (14.9)	900,007 (14.8)	79,159 (15.1)	14,551 (47.6)
	Hispanic	319,826 (4.9)	295,628 (4.9)	24,198 (4.6)	1,975 (6.5)
	Other	303,618 (4.6)	279,760 (4.6)	23,858 (4.5)	1,779 (5.8)
	Unknown	488,135 (7.4)	452,617 (7.5)	35,518 (6.8)	712 (2.3)
VACS Index 2.0 components*					
Age, years, Median (IQR)		65 (55–75)	65 (55–75)	64 (55–75)	55 (49–62)
	<50	1,131,735 (17.2)	1,046,926 (17.3)	84,809 (16.1)	8,537 (28.0)
	50–64	2,057,651 (31.2)	1,874,900 (30.9)	182,751 (34.8)	16,709 (54.7)
	65–79	2,309,838 (35.1)	2,140,653 (35.3)	169,185 (32.2)	5,017 (16.4)
	80+	1,089,464 (16.5)	1,000,453 (16.5)	89,011 (16.9)	276 (0.9)
CD4, No. (%)	<200				3,819 (12.5)
	200–499				11,194 (36.7)
	500+				15,526 (50.8)
HIV-RNA > 500 copies/ml, No. (%)					4,394 (14.4)
Hemoglobin (g/dl), Median (IQR)		14.4 (13.3–15.3)	14.4 (13.3–15.3)	14.3 (13.2–15.3)	14.2 (13.0–15.2)
FIB4, No. (%)	<1.45	3,508,814 (53.3)	3,243,588 (53.5)	265,226 (50.4)	16,442 (53.8)
	1.45–3.25	2,681,937 (40.7)	2,458,447 (40.5)	223,490 (42.5)	11,391 (37.3)
	>3.25	397,937 (6.0)	360,897 (6.0)	37,040 (7.0)	2,706 (8.9)
eGFR (ml/min), Median (IQR)		82 (65–96)	82 (65–95)	84 (67–97)	85 (68–100)
Hepatitis C infection		254,064 (3.9)	232,366 (3.8)	21,698 (4.1)	7,389 (24.2)
Albumin (g/dl), Median (IQR)		4.0 (3.8–4.3)	4.0 (3.8–4.3)	4.0 (3.8–4.3)	4.0 (3.7–4.3)
White blood count (k/ml), Median (IQR)		6.8 (5.6–8.2)	6.8 (5.6–8.2)	6.7 (5.5–8.1)	5.6 (4.5–7.0)
Body mass index, kg/m2, Median (IQR)		28.7 (25.4–32.6)	28.7 (25.4–32.6)	28.6 (25.3–32.5)	25.8 (22.8–29.4)
VACS Index 2.0, Score, Median (IQR)		69 (59–81)	69 (59–81)	69 (60–82)	47 (36–61)
Charlson Comorbidity Index **, No. (%)					
Myocardial infarction		118,723 (1.8)	108,953 (1.8)	9,770 (1.9)	541 (1.8)
Congestive heart failure		345,644 (5.2)	319,481 (5.3)	26,163 (5.0)	1,229 (4.0)
Peripheral vascular disease		355,255 (5.4)	326,920 (5.4)	28,335 (5.4)	987 (3.2)
Cerebrovascular disease		348,508 (5.3)	320,079 (5.3)	28,429 (5.4)	1,177 (3.9)
Dementia		107,067 (1.6)	99,417 (1.6)	7,650 (1.5)	297 (1.0)
Chronic pulmonary disease		867,328 (13.2)	797,133 (13.1)	70,195 (13.4)	3,227 (10.6)
Rheumatic disease		80,297 (1.2)	73,967 (1.2)	6,330 (1.2)	129 (0.4)
Peptic ulcer disease		52,132 (0.8)	47,780 (0.8)	4,352 (0.8)	204 (0.7)
Diabetes without chronic complications		1,309,211 (19.9)	1,200,014 (19.8)	109,197 (20.8)	3,738 (12.2)
Diabetes with chronic complications		354,847 (5.4)	328,682 (5.4)	26,165 (5.0)	1,043 (3.4)
Hemiplegia or paraplegia		37,536 (0.6)	34,553 (0.6)	2,983 (0.6)	233 (0.8)
Kidney disease, mild to moderate		344,915 (5.2)	314,917 (5.2)	29,998 (5.7)	2,251 (7.4)
Kidney disease, severe		92,492 (1.4)	86,256 (1.4)	6,236 (1.2)	879 (2.9)
Any malignancy		547,244 (8.3)	501,830 (8.3)	45,414 (8.6)	2,555 (8.4)
Metastatic solid tumor		42,914 (0.7)	39,550 (0.7)	3,364 (0.6)	251 (0.8)
Liver disease, mild		192,700 (2.9)	178,599 (2.9)	14,101 (2.7)	4,893 (16.0)
Liver disease, moderate to severe		24,159 (0.4)	22,155 (0.4)	2,004 (0.4)	268 (0.9)
Score	0	3,329,894 (50.5)	3,067,776 (50.6)	262,118 (49.9)	N/A
	1	1,590,282 (24.1)	1,460,791 (24.1)	129,491 (24.6)	N/A
	2	845,357 (12.8)	776,695 (12.8)	68,662 (13.1)	N/A
	3	420,629 (6.4)	386,490 (6.4)	34,139 (6.5)	15,811 (51.8)
	4	189,283 (2.9)	174,139 (2.9)	15,144 (2.9)	7,031 (23.0)
	5	90,585 (1.4)	83,461 (1.4)	7,124 (1.4)	3,542 (11.6)
	6 or greater	122,658 (1.9)	5,949,352 (98.1)	9,078 (1.7)	4,155 (13.6)

^*^VACS Index components used value closest to index date, 18 months before to 14 days after. ^**^Charlson Comorbidity Index used diagnoses documented by ICD diagnosis code in the 2 years prior to index date.

### Statistical analyses

#### Development and validation in the general PWoH VHA patients

Noting important demographic differences between our general patient sample and our prior samples in PWH, we considered whether associations between predictors and all-cause mortality were different by reassessing variable weights and model calibration. To provide a test of the temporal generalizability, we included those with an index visit between 2007–2011 and 2013–2017 in the developmental sample, withholding those identified in 2012 for inclusion in the independent validation sample. Choosing 2012 for the holdout sample for temporal external validation allowed us to ensure similar average follow-up time and still provide a test of generalizability. We developed a series of prognostic models to evaluate associations between predictors and all-cause mortality.

We used Cox proportional hazard models to predict 10-year mortality with predictors as follows: (1) age, (2) CCI components plus age, (3) VACS Index 2.0 score as published, (4) VACS Index 2.0 components (to allow reweighting, categorical values), (5) CCI components and VACS Index components (continuous values with appropriate functional forms), (6) #5 plus age by CCI interaction, (7) #6 plus sex. Model performance was assessed using Akaike’s information criterion (AIC, lower is better) for model fit and Harrell’s c-statistic (range 0.5–1.0, higher is better) for discrimination. Because of the extremely large sample size c-statistics were generated by averaging results from 5 sets of random samples of 100,000 observations (standard error was the same for all samples). Calibration (details below) was assessed by comparing to observed mortality. For models using continuous numeric variables extreme values were replaced with 1st or 99th percentile to avoid undue influence and variables were centered at clinically meaningful values for interpretability and to avoid variance inflation when using square and cubic terms necessary for using continuous measures.

For final candidate models [#5–#7], we translated regression output to meaningful risk scores that could be compared across models. For each variable, we multiplied regression coefficients by patient’s individual values, then summed to create a linear predictor (Xbeta). Then we scaled to 0–100 by dividing each patient’s Xbeta by the difference of highest and lowest values across all patients in the development sample. We then predicted ten-year all-cause mortality using the newly derived risk score as the only predictor in a parametric (gamma) survival regression model. Observed mortality was estimated using the Kaplan–Meier (KM) method. For each five-point interval of score we calculated mortality and 95% confidence intervals (CI). Calibration was assessed by overlaying plots of observed mortality on predicted mortality and checking for systematic deviations.

We summarized risk scores derived from the final model [#7], dubbed VACS-CCI, and assessed performance in development and validation samples, overall and in subgroups (men: non-Hispanic white, non-Hispanic black, Hispanic, age < 65, age 65+; women; level of CCI score). Risk scores were summarized with mean, standard deviation, and coefficient of variation (COV), which measures the spread of values relative to the magnitude of the mean. We assessed discrimination and calibration as described above. Data points of observed mortality were plotted when there was a minimum of 10 deaths and 5 survivors for a given level of risk score.

Finally, using the full data set for maximum precision, we predicted one-, two-, five-, and ten-year mortality again using the gamma survival model with VACS-CCI score as the only predictor. Upon publication, the score calculation and mortality prediction will be available online via MDCalc. We will also collaborate with VHA leadership to provide automatic calculation in the VHA EHR.

#### VACS Index and VACS-CCI in PWH

VACS-CCI and VACS Index 2.0 were calculated for each person at their index date. We used Cox proportional hazard models to predict 10-year mortality with predictors as follows: (1) age, (2) CCI components plus age, (3) VACS Index 2.0 score as published (add reference), (4) VACS-CCI as created above, (5) VACS-CCI plus CD4 and HIV-RNA viral load (VL). Model performance was assessed using Akaike’s information criterion (AIC, lower is better) for model fit and Harrell’s C-statistic (range 0.5–1.0, higher is better) for discrimination. Additionally, 10-year mortality predictions based on VACS Index and VACS-CCI were compared graphically with observed mortality for CD4 (<200, 200–499, 500+) and HIV RNA-1 VL subgroups. Data points of observed mortality were plotted when there was a minimum of 10 deaths and 5 survivors for a given level of risk score. We also compared prevalence of comorbid disease groups from the CCI by CD4 groups (<200, 200–499, 500+) using tests for trend.

## Results

### Patient characteristics in the general PWoH and PWH VHA patients

We identified 6,062,932 PWoH for development (years 2007–2011 and 2013–2017), 525,756 for validation (year 2012), and 30,539 PWH (years 2007–2017) (Table 1). Demographics, laboratory values, and frequency of comorbidities comprising the CCI were similar between the development and validation samples. Compared to the sample of 6,588,688 PWoH in the development and validation samples, the PWH sample was younger (median age 55 vs. 65 years), less likely to be female (3% vs. 6%), more likely to be non-Hispanic Black (48% vs. 15%), had lower median VACS Index score (47 vs. 69), had a higher proportion with elevated FIB-4, HCV, other liver disease as defined by CCI, and lower proportion with diabetes. Over half of the randomly selected index dates were in 2013–2017 for the development and validation (52%) and PWH (55%) samples. Median observation time was 6.2 years (IQR = 4.4–8.8) in development, 9.4 years (IQR = 5.5–9.8) in validation, and 6.5 years (IQR = 4.6–9.5) in PWH. After a maximum of 10 years follow-up there were 2,082,928 deaths in development, 203,953 in validation, and 9,005 in PWH. Additional characteristics are also shown in Table 1.

### Model development in the general PWoH VHA patients

**Figure 1:**
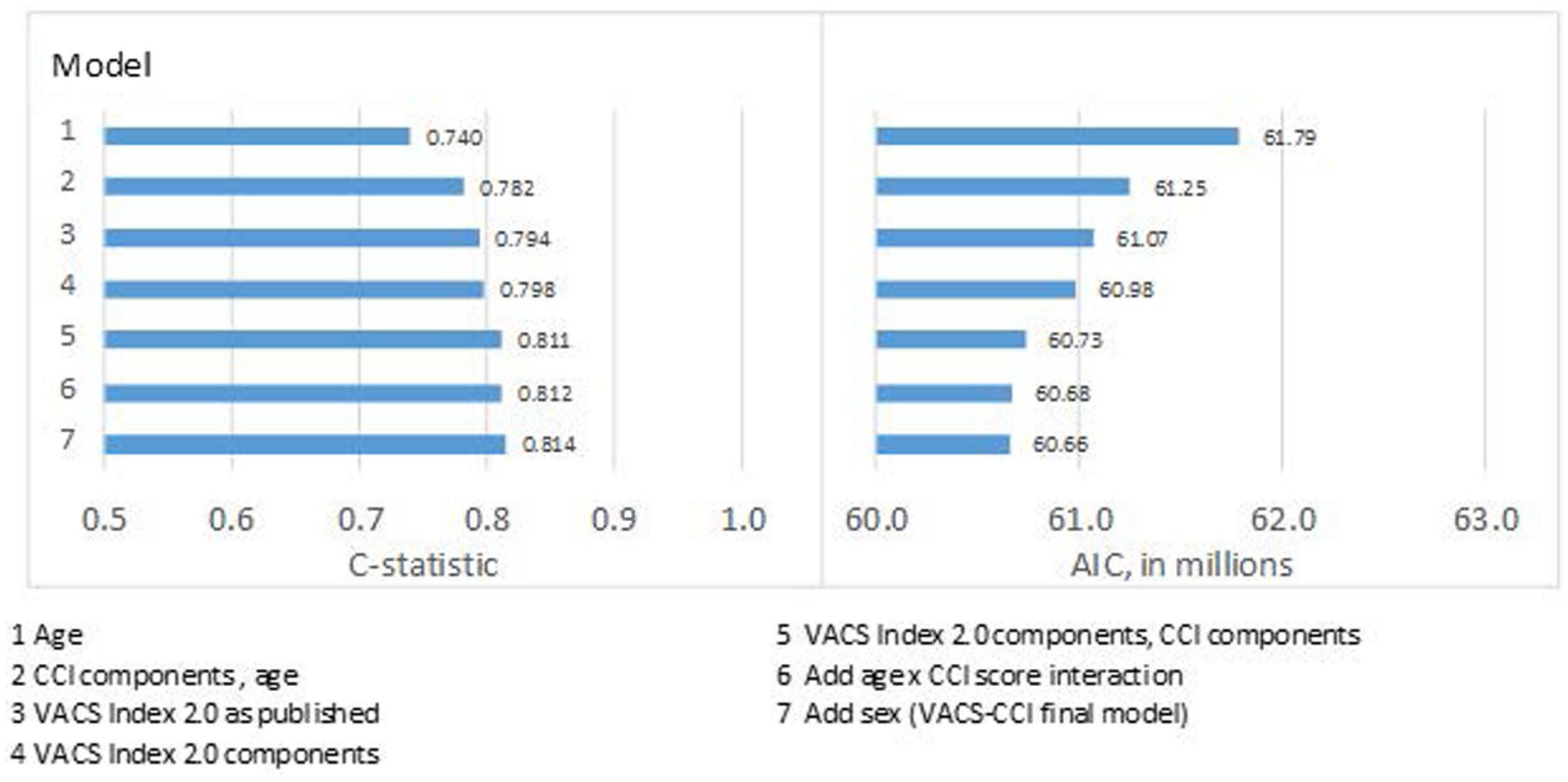
Model fit (AIC) and discrimination (c-statistic) from series of models for 10-year, all-cause mortality using Cox regression in development sample.

Compared to age alone (C-statistic = 0.740), discrimination for predicting 10-year mortality in the development dataset was better for CCI components plus age (C-statistic = 0.782), the VACS Index 2.0 as published (C-statistic = 0.794), and VACS Index 2.0 components reweighted (C-statistic = 0.798) (Figure 1). Combining CCI and VACS index components (using either categorical or continuous values) improved discrimination further (C-statistic = 0.811). Plots of mortality vs. risk score revealed poor calibration at extreme scores in younger patients and women. This was resolved by adding an age x CCI score interaction and sex to the model. The final VACS-CCI model (Table 2, Model 7) had the highest C-statistic (C-statistic = 0.814). Similar patterns of improvement were seen with AIC which dropped from 61,790 for CCI plus age to 60,660 for VACS-CCI.

**Table 2:** VACS-CCI prognostic model developed in 6,062,932 U.S veterans with index visit in 2007–2011 or 2013–2017.

Parameter	Value	Parameter Estimate	c^2^	*p*	Hazard Ratio	95% Confidence Interval
Sex	Female vs. Male	−0.513	10,632	<0.0001	0.60	(0.59–0.60)
VACS Index components*						
Age	*X* = age – 50	0.278	17,800	<0.0001	1.32	(1.32–1.33)
	*X* ^2^	0.006	168	<0.0001	1.01	(1.01–1.01)
	*X* ^3^	−0.001	185	<0.0001	1.00	(1.00–1.00)
Hemoglobin	*X* = hemoglobin – 14	−0.115	31,380	<0.0001	0.89	(0.89–0.89)
	*X* ^2^	0.024	13,337	<0.0001	1.03	(1.02–1.03)
	*X* ^3^	0.004	6,899	<0.0001	1.00	(1.00–1.00)
FIB4	*X*	0.205	16,017	<0.0001	1.23	(1.22–1.23)
	*X* ^2^	−0.006	1,118	<0.0001	0.99	(0.99–0.99)
eGFR	*X* = egfr – 90	0.071	8,831	<0.0001	1.07	(1.07–1.08)
	*X* ^2^	0.036	16,170	<0.0001	1.04	(1.04–1.04)
	*X* ^3^	0.002	3,064	<0.0001	1.00	(1.00–1.00)
Albumin	*X* = albumin – 4	−0.405	30,915	<0.0001	0.67	(0.66–0.67)
	*X* ^2^	0.326	10,439	<0.0001	1.39	(1.38–1.39)
	*X* ^3^	0.156	4,604	<0.0001	1.17	(1.16–1.17)
WBC	*X* = WBC – 4	−0.011	33	<0.0001	0.99	(0.99–0.99)
	*X* ^2^	0.053	3,011	<0.0001	1.05	(1.05–1.06)
	*X* ^3^	−0.008	2,042	<0.0001	0.99	(0.99–0.99)
	X^4^	0.000	1,242	<0.0001	1.00	(1.00–1.00)
BMI	*X* = BMI – 25	−0.061	94,361	<0.0001	0.94	(0.94–0.94)
	*X* ^2^	0.005	23,742	<0.0001	1.01	(1.01–1.01)
	*X* ^3^	0.000	3,987	<0.0001	1.00	(1.00–1.00)
Hepatitis C infection		0.273	4,487	<0.0001	1.31	(1.30–1.33)
Charlson Comorbidity Index**						
Myocardial infarction		0.008	4	0.0444	1.01	(1.00–1.02)
Congestive heart failure		0.420	28,087	<0.0001	1.52	(1.52–1.53)
Peripheral vascular disease		0.141	3,012	<0.0001	1.15	(1.15–1.16)
Cerebrovascular disease		0.114	1,869	<0.0001	1.12	(1.11–1.13)
Dementia		0.489	16,977	<0.0001	1.63	(1.62–1.64)
Chronic pulmonary disease		0.279	17,229	<0.0001	1.32	(1.32–1.33)
Rheumatic disease		−0.034	37	<0.0001	0.97	(0.96–0.98)
Peptic ulcer disease		−0.074	140	<0.0001	0.93	(0.92–0.94)
Diabetes without chronic complications		0.053	588	<0.0001	1.05	(1.05–1.06)
Diabetes with chronic complications		0.075	498	<0.0001	1.08	(1.07–1.09)
Hemiplegia or paraplegia		−0.049	40	<0.0001	0.95	(0.94–0.97)
Kidney disease, mild to moderate		−0.199	4,622	<0.0001	0.82	(0.82–0.82)
Kidney disease, severe		−0.098	346	<0.0001	0.91	(0.90–0.92)
Any malignancy		0.090	840	<0.0001	1.10	(1.09–1.10)
Metastatic solid tumor		1.184	35,592	<0.0001	3.27	(3.23–3.31)
Liver disease, mild		−0.045	92	<0.0001	0.96	(0.95–0.97)
Liver disease, moderate to severe		0.226	647	<0.0001	1.25	(1.23–1.28)
Interaction of Charlson Comorbidity Index and age						
CCI	Age					
0	<50	−0.698	4,504	<0.0001	0.50	(0.49–0.51)
	50–64	−0.386	7,581	<0.0001	0.68	(0.67–0.69)
	65–79	−0.407	6,687	<0.0001	0.67	(0.66–0.67)
	80+	−0.125	412	<0.0001	0.88	(0.87–0.89)
1	<50	−0.158	170	<0.0001	0.85	(0.83–0.87)
	50–64				Ref	(0.00–0.00)
	65–79	−0.102	470	<0.0001	0.90	(0.89–0.91)
	80+	−0.047	60	<0.0001	0.95	(0.94–0.97)
2	<50	0.388	592	<0.0001	1.47	(1.43–1.52)
	50–64	0.271	3,056	<0.0001	1.31	(1.30–1.32)
	65–79	0.036	51	<0.0001	1.04	(1.03–1.05)
	80+	−0.037	33	<0.0001	0.96	(0.95–0.98)
3+	<50	0.878	3,100	<0.0001	2.41	(2.33–2.48)
	50–64	0.569	11,375	<0.0001	1.77	(1.75–1.79)
	65–79	0.192	1,111	<0.0001	1.21	(1.20–1.23)
	80+	−0.088	163	<0.0001	0.92	(0.90–0.93)

### VACS-CCI risk score summary, discrimination, and calibration in the general PWoH VHA patients

Using the VACS-CCI risk score derived from Model 7 as the only predictor (1 variable rather than individual components), we observed minimal degradation of discrimination in development (C-statistic = 0.813) or validation (C-statistic = 0.810) datasets compared to the original (Model #7, C-statistic = 0.814). Observed 1-, 5-, and 10-year mortality showed good concordance with predicted mortality in development and validation datasets (Supplementary Figure S1). The exception was 1-year and 5-year mortality in those with scores greater than 70 (<0.5%), where the model over-predicted mortality. For context the mean VACS-CCI was 37 (SD = 13) in both the development and validation datasets.

**Figure 2:**
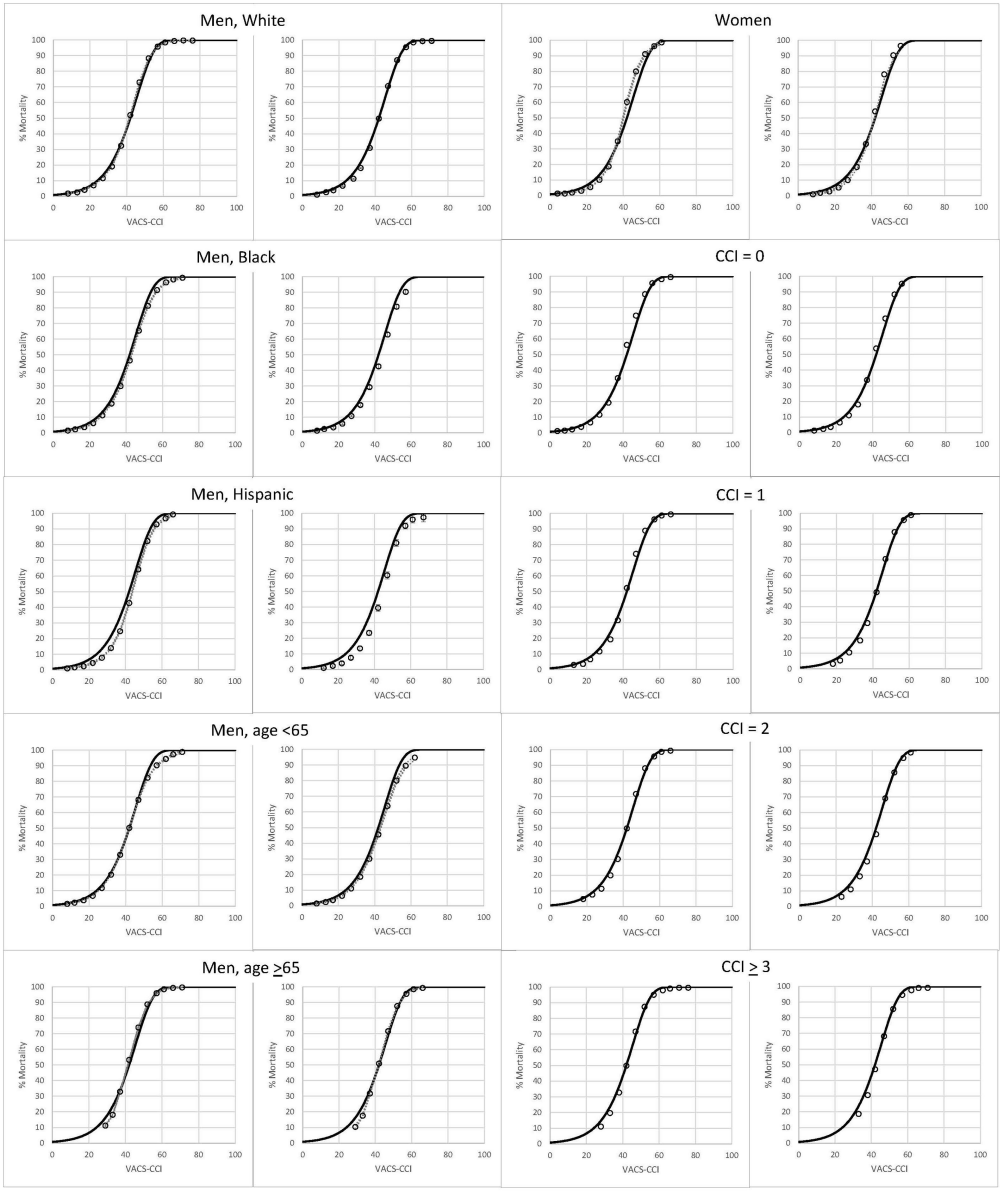
Observed (open circles) and predicted (solid line) 10-year, all-cause mortality as a function of VACS-CCI score. 95% confidence intervals for observed mortality are very narrow and may be difficult to discern.

In subgroup analysis there were more than 22,000 observations and 4,000 deaths in all groups, even among the smallest groups (Hispanics and women) in the validation dataset (Table 3). The proportion that died during 10 years of follow-up ranged from 11 to 74%; and was lowest in women and those under age 65, and highest in those with CCI scores of 3 or greater. Variation within subgroups was highest among women (COV = 49%) and considerably lower in the age 65 and older subgroup (COV = 20%) and those with higher CCI scores. Within subgroups, C-statistics were similar in development and validation samples. As expected, C-statistics were smaller when there was less variation in a subgroup. For example, for CCI scores of 0, 1, 2 and ≥ 3, C-statistics were 0.819, 0.767, 0.736, and 0.714, respectively (Supplementary Figure S2). Observed mortality was congruent with predicted mortality (from VACS-CCI) among all subgroups examined for both the development and validation datasets (Figure 2).

**Table 3:** VACS-CCI summary statistics, and discrimination of 10-year mortality using VACS-CCI score as only predictor in a Cox model, in development and validation samples, overall and by subgroup.

Subgroup		*N*	Deaths	% Died	VACS-CCI score		Discrimination
					Mean (SD)	% COV	c-statistic (95% CI)
Development							
Overall		6,062,932	2,082,928	34	36.6 (13.3)	36	0.813 (0.811, 0.815)
Men	White	3,924,624	1,431,524	36	38.1 (12.5)	33	0.800 (0.798, 0.802)
	Black	794,185	199,501	25	33.7 (13.3)	40	0.822 (0.819, 0.825)
	Hispanic	276,001	65,441	24	33.4 (13.9)	42	0.850 (0.847, 0.852)
	Age < 65	2,600,537	446,905	17	28.5 (10.7)	38	0.798 (0.794, 0.801)
	Age ≥ 65	3,086,015	1,593,157	52	45.1 (9.1)	20	0.756 (0.753, 0.758)
Women		376,380	42,866	11	23.2 (11.5)	49	0.871 (0.868, 0.875)
CCI	0	3,067,776	616,957	20	29.5 (11.9)	40	0.819 (0.816, 0.822)
	1	1,460,791	558,727	38	39.8 (9.7)	24	0.767 (0.765, 0.769)
	2	776,695	385,494	50	44.5 (8.5)	19	0.736 (0.734, 0.739)
	3+	757,670	521,750	69	51.3 (8.7)	17	0.714 (0.712, 0.716)
Validation							
Overall		525,756	203,953	39	36.9 (13.0)	35	0.810 (0.808, 0.812)
Men	White	345,228	144,302	42	38.4 (12.2)	32	0.797 (0.795, 0.799)
	Black	70,583	20,605	29	33.8 (13.1)	39	0.811 (0.808, 0.814)
	Hispanic	22,819	6,371	28	34.1 (13.5)	40	0.839 (0.834, 0.844)
	Age < 65	241,165	48,799	20	29.6 (10.6)	36	0.782 (0.780, 0.784)
	Age ≥ 65	253,756	150,783	59	45.4 (9.1)	20	0.749 (0.748, 0.750)
Women		30,835	4,371	14	23.7 (11.5)	49	0.863 (0.857, 0.869)
CCI	0	262,118	63,042	24	29.9 (11.8)	39	0.819 (0.817, 0.820)
	1	129,491	55,217	43	39.8 (9.6)	24	0.767 (0.765, 0.769)
	2	68,662	37,348	54	44.4 (8.5)	19	0.735 (0.732, 0.738)
	3+	65,485	48,346	74	51.2 (8.7)	17	0.717 (0.714, 0.719)

### Translating the VACS-CCI into mortality risk estimates in the general PWoH VHA patients

**Figure 3:**
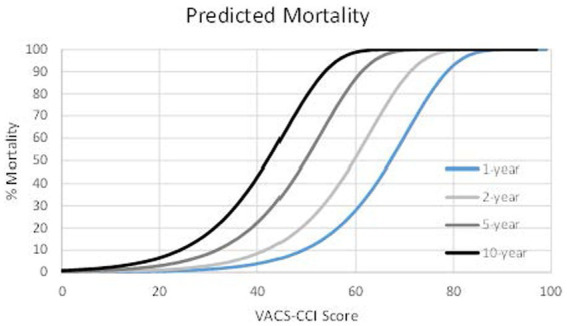
VACS-CCI predicted mortality based on general (PWoH) sample, 2007–2017.

In the combined development and validation samples VACS-CCI score was approximately normally distributed with mean 36.6 (SD 13.2) and median 37 (IQR 28–46); 80% of scores were between 17 and 54; 90% of scores were between 14 and 58. Predicted mortality in this sample (index visit 2007–2017) ranged from 0 to 100% for one-, two-, five-, and ten-year mortality (Figure 3; Supplementary Table S3). For example, for VACS-CCI score of 30, predicted mortality was 1% at 1 year, 3% at 2 years, 8% at 5 years, and 18% at 10 years. For a score of 45, predicted mortality was 6% at 1 year, 14% at 2 years, 35% at 5 years, and 61% at 10 years. Around the median score, a one-point increment in VACS-CCI score translated to approximately 10% increase in predicted mortality.

### VACS-CCI validation in PWH

Model discrimination for predicting 10-year mortality in the sample of PWH was better for the CCI components plus age (C-statistic = 0.699), VACS Index 2.0 as published (C-statistic = 0.774), VACS-CCI (C-statistic = 0.772) and the VACS-CCI plus CD4 and HIV RNA-1 VL (C-statistic = 0.785) compared to age alone (C-statistic = 0.637).

**Figure 4:**
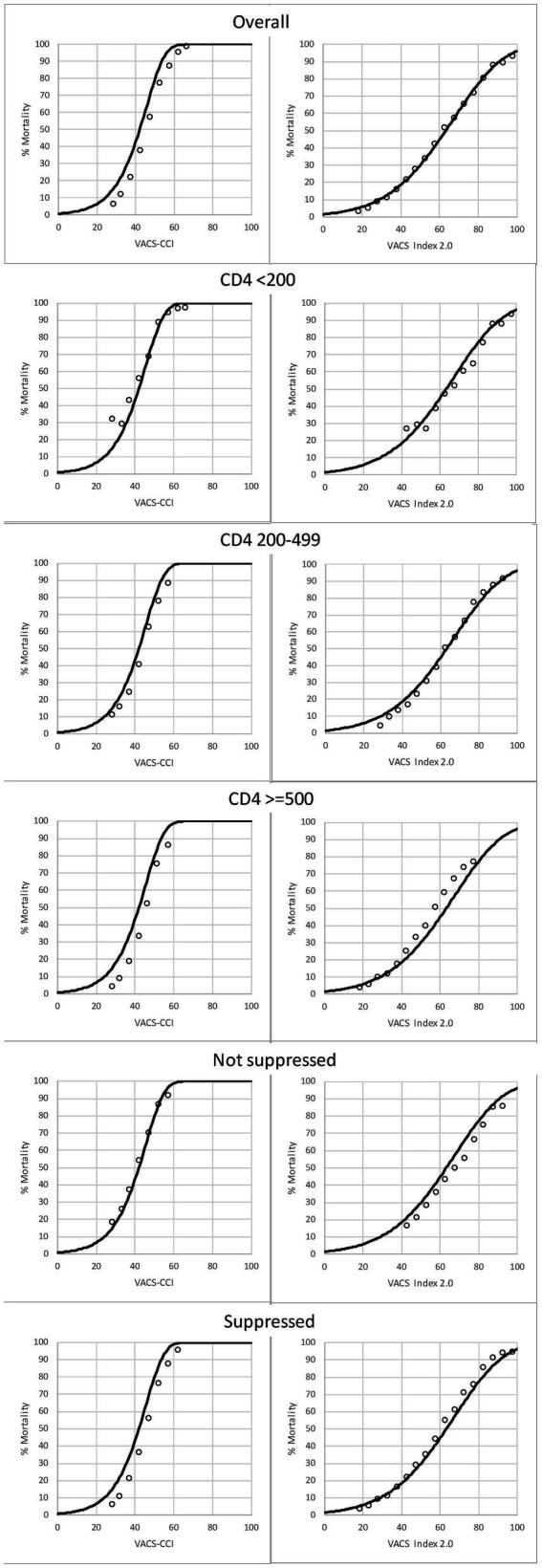
Among PWH, observed (open circles) and predicted (solid line) 10-year, all-cause mortality as a function of VACS-CCI and VACS Index 2.0 risk scores. 95% confidence intervals for observed mortality are very narrow and may be difficult to discern.

Plots of observed and predicted 10-year mortality (using both VACS Index and VACS-CCI to predict mortality) overall and by CD4 and HIV-RNA VL groups are shown in Figure 4. Observed mortality was generally congruent with predicted mortality among subgroups, including for PWH with CD4 < 200.

**Figure 5:**
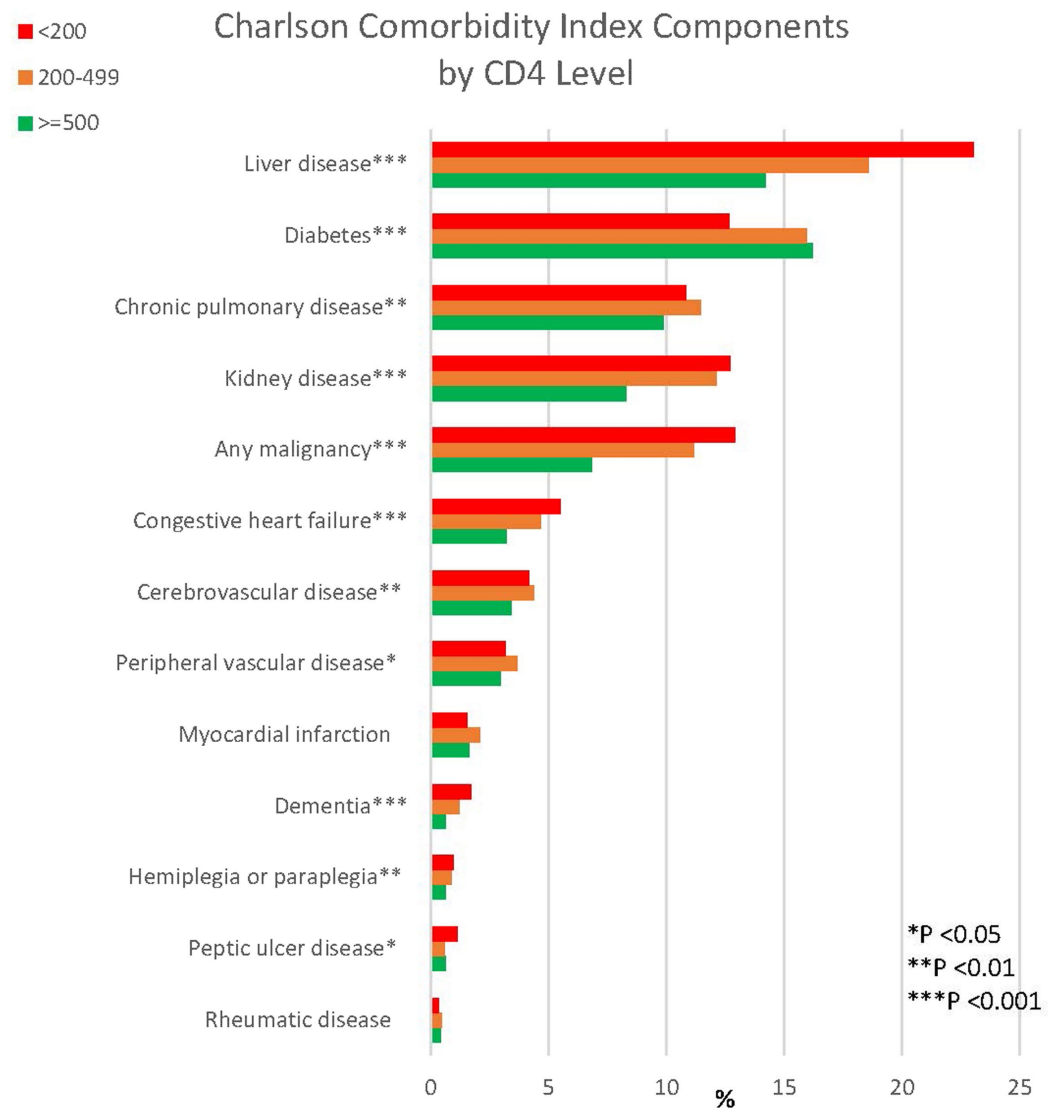
Charlson comorbidity components by CD4 level among PWH.

To better understand PWH with CD4 < 200, we compared conditions included in the CCI by CD4 count groups (<200, 200–499, 500+). Those with CD4 < 200 were more likely than those with higher CD4 to have many comorbidities including liver disease, kidney disease, malignancy, congestive heart failure, and dementia (*p*-trend <0.0001 for all, Figure 5).

## Discussion

Among PWH and PWoH in VHA care, age alone weakly discriminates risk of mortality, and both VACS Index and the newly developed VACS-CCI provide much better 10-year mortality discrimination. Additionally, the VACS-CCI, which includes components of the VACS Index plus comorbidities included in the CCI, was more consistently calibrated than the VACS Index among important subgroups.

The VACS-CCI may be an important improved version of the VACS Index which has been widely used in HIV clinical research as an estimate of overall disease burden and in clinical settings to prioritize health screening and medical management. For example, colon cancer screening and active treatment for non-metastatic prostate cancer are both indicated in those expected to live more than 10 years. In contrast, end-of-life planning should be considered among those with a shorter life expectancy (7).

There are notable limitations to both the CCI and VACS Index components. For CCI, comorbidities are considered to be present only if the corresponding diagnostic code is present. While this is an inclusive approach, it can result in under estimation of risk for those with fewer clinical encounters. For example, a recent study of CCI as a predictor of mortality after COVID-19 found that CCI based on ICD codes assigned during a single inpatient stay substantially under-estimated risk of mortality compared to also including codes assigned at outpatient visits within VHA (28). In contrast, VACS Index is a less inclusive approach as it requires biomarkers to be present with only two assumptions and allowances regarding missing data. Missing values are assumed normal when only albumin is missing, or if only ALT or AST are missing. In the general sample of people engaged in care, 19% of otherwise eligible patients were excluded due to missing laboratory data. Future work will explore developing and validating algorithms for more systematic estimation of other missing values to minimize the proportion of patients excluded.

In addition to the issue of missing laboratory data, there were several other noteworthy limitations. First, while the VACS-CCI is ready for application within the national VA and has demonstrated accuracy overall and among 979,166 patients who are Black, 407,215 women, and 319,826 patients of Hispanic ethnicity, it will require validation outside VA prior to external application. Our prior experience with validating VACS Index among external samples of people with HIV suggests that this is likely to be successful. Second, additional work will be needed to help clinicians understand the major drivers of an individual patient’s risk and which of these might be modifiable. Similarly, if VACS-CCI is to be used as an outcome, we will need to explore the degree to which changes in estimated risk correspond to actual changes in individual risk. This need to reflect responsiveness to change is supported by our use of a random visit baseline, thus ensuring a diversity of observations with respect to stage of disease and age. Third, mental health, alcohol and substance use, and socio-economic factors are not included in the VACS-CCI. Nevertheless, we believe that medical harm from these factors typically operates through variables already included in VACS-CCI. For example, we have previously demonstrated that people who have unhealthy alcohol use, use injection drugs, or smoke cigarettes have higher VACS Index scores (15, 29). Lastly, although the calibration of VACS-CCI for Hispanic patients appeared to over-estimate mortality risk, we have not included race or ethnicity as a variable in the VACS-CCI. This decision is based on recommendations from multiple sources regarding predictive tools (30, 31). and inclusion of race would complicate the use of VACS-CCI internationally.

This study has many strengths. We included only variables that would be widely available in clinical practice and research databases and could also be accurately and reliably measured. The very large development and validation sample sizes allowed us to refine VACS-CCI to perform well in important subgroups, including women. Although only 6% of the sample, 400,000 women provided adequate power for us to discern poor calibration, which was resolved by adjusting for female sex. We found that the discriminating power could be enhanced by including both CCI score and indicators for the individual conditions. We also discovered an interesting interaction between age and CCI score in which for those with low CCI scores (0 or 1), greater age increased the associated HR whereas for those with higher CCI scores (3 or 4), greater age decreased the associated HR. In other words, when you have few serious comorbidities, increasing age is an important driver of prognosis. When you have several serious comorbidities, the associated increased risk of mortality is greater when you are younger. VACS-CCI will be made accessible for clinical and research purposes in the following ways. We will post the programming to calculate the score on GitHub. We will also collaborate with MDCalc to post a web-based calculator as with VACS Index 2.0 (see Footnote 1). Further, MDCalc is developing a direct implementation in electronic health record-based decision support systems which would obviate the need to enter data.

Among PWH, by comparing CCI components by CD4 groups, we found that conditions not captured by the VACS Index, such as malignancy, congestive heart failure, and dementia, were more common among people with CD4 < 200. Additionally, liver disease and kidney disease were more common among people with CD4 < 200; while liver and kidney disease would be captured by FIB-4 and eGFR, symptom and disease severity not reflected in lab values may be further captured by ICD diagnoses and may be important additional predictors of mortality. The dose response association of common comorbid diseases with lower CD4 count underscores the likely role of HIV in non-AIDS conditions and warrants further exploration.

Age alone should not be used to determine health screening, treatment or other recommendations for patients with and without HIV. VACS-CCI provides excellent discrimination and calibration for 10-year all-cause mortality in a general sample of over six million patients and over 30,000 PWH in VHA care. VACS-CCI is ready for application in clinical research and patient care within the general VHA patient population but will require external validation prior to external use. Future work will also include adding CD4 count and HIV RNA to the VACS-CCI for PWH and validating it in independent data.

## Data availability statement

The data that support the findings of this study are not permitted to leave the VA firewall without a Data Use Agreement due to VA regulations. However, VA data are made freely available to researchers with an approved VA study protocol. For more information, please contact the VA Information Resource Center (VIReC) at VIRec@va.gov or the corresponding author.

## Ethics statement

The studies involving humans were approved by Yale University and VA Connecticut Healthcare System institutional review boards. The studies were conducted in accordance with the local legislation and institutional requirements. The ethics committee/institutional review board waived the requirement of written informed consent for participation from the participants or the participants’ legal guardians/next of kin because the study utilized electronic health record data; there was no direct patient contact.

## Author contributions

KM: Conceptualization, Methodology, Writing – original draft, Writing – review & editing, Resources. AJ: Conceptualization, Funding acquisition, Investigation, Methodology, Resources, Supervision, Validation, Writing – original draft, Writing – review & editing. VM: Conceptualization, Investigation, Methodology, Resources, Writing – review & editing. MR-B: Conceptualization, Investigation, Resources, Writing – review & editing. RH: Data curation, Investigation, Resources, Writing – review & editing. KO: Conceptualization, Investigation, Resources, Writing – review & editing. SB: Conceptualization, Investigation, Resources, Writing – review & editing. KB: Funding acquisition, Investigation, Resources, Writing – review & editing. JT: Conceptualization, Data curation, Formal analysis, Methodology, Supervision, Validation, Visualization, Writing – review & editing.

## Footnotes

^1^ https://www.mdcalc.com/calc/10402/veterans-aging-cohort-study-vacs-2.0-index
