# Corrigendum: Combining Charlson comorbidity and VACS indices improves prognostic accuracy for all-cause mortality for patients with and without HIV in the Veterans Health Administration

**DOI:** 10.3389/fmed.2024.1532350

**Published:** 2025-01-08

**Authors:** Kathleen A. McGinnis, Amy C. Justice, Vincent C. Marconi, Maria C. Rodriguez-Barradas, Ronald G. Hauser, Krisann K. Oursler, Sheldon T. Brown, Kendall J. Bryant, Janet P. Tate

**Affiliations:** ^1^VA Connecticut Healthcare System, West Haven, CT, United States; ^2^Yale School of Medicine, New Haven, CT, United States; ^3^The Atlanta Veterans Affairs Medical Center, Emory University School of Medicine and Rollins School of Public Health, Atlanta, GA, United States; ^4^VA Medical Center, Decatur, GA, United States; ^5^Infectious Diseases Section, Michael E. DeBakey Veterans Affairs Medical Center, Houston, TX, United States; ^6^Department of Medicine, Baylor College of Medicine, Houston, TX, United States; ^7^Department of Laboratory Medicine, Yale University School of Medicine, New Haven, CT, United States; ^8^Department of Internal Medicine, Virginia Tech Carilion School of Medicine, Roanoke, VA, United States; ^9^VA Salem Healthcare System, Salem, VA, United States; ^10^James J. Peters VA Medical Center, Bronx, NY, United States; ^11^National Institute on Alcohol Abuse and Alcoholism, Bethesda, MD, United States

**Keywords:** VACS Index, Charlson Comorbidity Index, HIV, mortality, prediction

In the published article, there was an error in the CD4 < 200 plot in Figure 4 and the corresponding interpretation. The corrected Figure 4 and its caption appear below.

**Figure 4:**
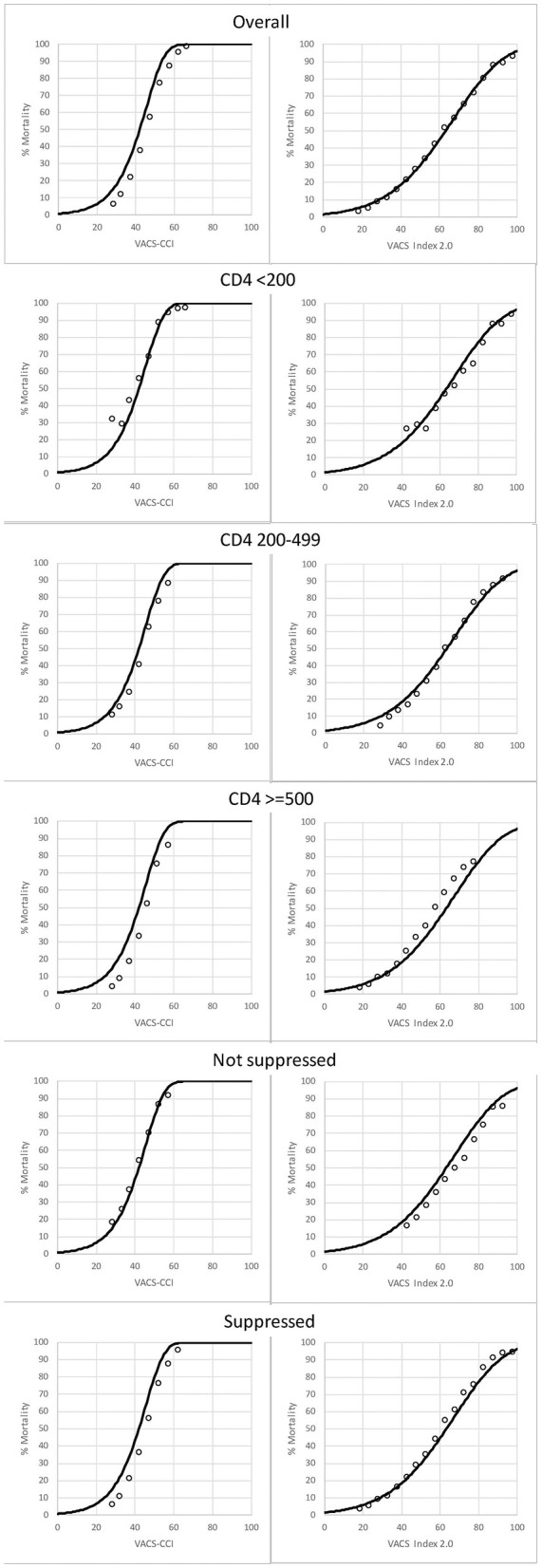
Among PWH, observed (open circles) and predicted (solid line) 10-year, all-cause mortality as a function of VACS-CCI and VACS Index 2.0 risk scores. 95% confidence intervals for observed mortality are very narrow and may be difficult to discern.

A correction has been made to **Results**, *VACS-CCI validation in PWH*, Paragraph 2.

This sentence previously stated:

“Observed mortality was generally congruent with predicted mortality among subgroups, except that VACS Index underestimated mortality for PWH with CD4 < 200.”

The corrected sentence appears below:

“Observed mortality was generally congruent with predicted mortality among subgroups, including for PWH with CD4 < 200.”

A correction has been made to **Results**, *VACS-CCI validation in PWH*, Paragraph 3.

This sentence previously stated:

“To better understand the underestimated mortality for PWH CD4 < 200 using VACS Index, we compared conditions included in the CCI by CD4 count groups (<200, 200–499, 500+).”

The corrected sentence appears below:

“To better understand PWH with CD4 < 200, we compared conditions included in the CCI by CD4 count groups (<200, 200–499, 500+).”

The authors apologize for these errors and state that this does not change the scientific conclusions of the article in any way. The original article has been updated.

