# Supplementary material for: Combining Charlson comorbidity and VACS indices improves prognostic accuracy for all-cause mortality for patients with and without HIV in the Veterans Health Administration
Source: Front Med (Lausanne). 2024 Jan 31;10:1342466. doi: 10.3389/fmed.2023.1342466 (PMC10864663; doi:10.3389/fmed.2023.1342466)
Supplement: Supplementary file 1 [file Table_1.pdf]

Supplementary Table S1. Stop Code Description

323	Primary care	6,133,839	93%
322	Women's clinic	118,452	2%
301	General internal medicine	93,340	1%
350	Geriatric primary care	62,463	1%
310	Infectious Disease	19,996	0.3%
303	Cardiology	18,156	0.3%
315	Neurology	17,540	0.3%
316	Oncology	12,737	0.2%
307	Gastroenterology	12,733	0.2%
120	Health screening	10,892	0.2%
312	Pulmonary	10,020	0.2%
313	Nephrology	8,356	0.1%
308	Hematology	8,012	0.1%
305	Endocrinology	7,152	0.1%
314	Rheumatology/Arthritis	6,601	0.1%
318	Geriatric clinic	6,286	0.1%
419	Anesthesia consult	4,711	0.1%
337	Hepatology	4,593	0.1%
160	Clinical pharmacy	3,741	0.1%
404	Gynecology	3,701	0.1%
999	Occupational health	3,481	0.1%
309	Hypertension	3,436	0.1%
306	Diabetes	3,380	0.1%
319	Geriatric evaluation and management	3,200	0.0%
420	Pain clinic	3,101	0.0%
415	Vascular surgery	3,018	0.0%
432	Pre-surgery evaluation	1,455	0.0%
201	Physical medicine and rehabilitation	1,287	0.0%
311	Pacemaker	1,270	0.0%
531	Mental health primary care	945	0.0%
302	Allergy/ Immunology	770	0.0%
40	Rheumatology/Arthritis	13	0.0%
99	Employee Health	6	0.0%
29	Cardiology	5	0.0%
		6,588,688	
