# Supplementary material for: Combining Charlson comorbidity and VACS indices improves prognostic accuracy for all-cause mortality for patients with and without HIV in the Veterans Health Administration
Source: Front Med (Lausanne). 2024 Jan 31;10:1342466. doi: 10.3389/fmed.2023.1342466 (PMC10864663; doi:10.3389/fmed.2023.1342466)
Supplement: Supplementary file 3 [file Table_3.pdf]

Supplementary Table S3. Predicted mortality in full sample (2007-2017) using VACS-CCI score as the only predictor in a gamma regression model

Score	N	1-year	2-year	5-year	10-year	Score	N	1-year	2-year	5-year	10-year
0	1	0.05	0.12	0.4	0.8						
1	17	0.06	0.13	0.4	0.9	51	122,092	11.9	24.6	55.2	82.7
2	160	0.07	0.15	0.4	1.0	52	113,852	13.2	26.9	58.9	85.7
3	486	0.07	0.17	0.5	1.1	53	104,959	14.5	29.4	62.8	88.4
4	1,138	0.08	0.19	0.5	1.2	54	95,081	16.0	32.1	66.6	90.8
5	2,433	0.09	0.21	0.6	1.3	55	85,907	17.6	34.9	70.4	92.9
6	5,315	0.10	0.2	0.7	1.5	56	76,557	19.4	38.0	74.1	94.7
7	11,808	0.11	0.3	0.7	1.7	57	67,162	21.3	41.1	77.6	96.1
8	21,290	0.13	0.3	0.8	1.8	58	58,425	23.4	44.5	81.0	97.3
9	32,041	0.14	0.3	0.9	2.1	59	50,749	25.6	48.0	84.1	98.2
10	42,713	0.16	0.4	1.0	2.3	60	43,295	28.0	51.6	87.0	98.8
11	52,803	0.18	0.4	1.1	2.5	61	36,810	30.6	55.3	89.6	99.3
12	63,768	0.20	0.4	1.3	2.8	62	30,760	33.4	59.1	91.8	99.6
13	73,697	0.2	0.5	1.4	3.1	63	25,828	36.3	62.9	93.8	99.8
14	83,233	0.2	0.5	1.6	3.5	64	21,373	39.4	66.7	95.4	99.9
15	90,180	0.3	0.6	1.7	3.9	65	17,686	42.7	70.5	96.7	99.9
16	93,090	0.3	0.7	1.9	4.3	66	14,620	46.1	74.2	97.7	100.0
17	89,085	0.3	0.7	2.2	4.8	67	11,943	49.7	77.7	98.5	100.0
18	83,223	0.4	0.8	2.4	5.3	68	9,526	53.3	81.1	99.0	100
19	77,561	0.4	0.9	2.7	5.9	69	7,763	57.1	84.2	99.4	100
20	74,744	0.5	1.0	3.0	6.5	70	6,102	60.9	87.1	100	100
21	76,527	0.5	1.1	3.3	7.2	71	4,992	64.7	89.7	100	100
22	80,302	0.6	1.3	3.7	8.0	72	3,830	68.5	91.9	100	100
23	86,321	0.6	1.4	4.1	8.8	73	3,063	72.3	93.9	100	100
24	93,189	0.7	1.6	4.5	9.8	74	2,399	75.9	95.5	100	100
25	102,247	0.8	1.8	5.0	10.8	75	1,830	79.3	96.7	100	100
26	111,625	0.9	2.0	5.6	12.0	76	1,444	82.6	97.8	100	100
27	123,440	1.0	2.2	6.2	13.2	77	1,117	85.6	98.5	100	100
28	139,329	1.1	2.4	6.8	14.6	78	842	88.3	99.0	100	100
29	156,173	1.2	2.7	7.6	16.1	79	660	90.8	99.4	100	100
30	172,965	1.3	3.0	8.4	17.7	80	503	92.9	100	100	100
31	186,045	1.5	3.3	9.3	19.5	81	373	94.6	100	100	100
32	195,934	1.7	3.7	10.3	21.4	82	273	96.1	100	100	100
33	203,151	1.8	4.1	11.3	23.5	83	205	97.2	100	100	100
34	207,426	2.1	4.5	12.5	25.7	84	156	98.1	100	100	100
35	209,888	2.3	5.0	13.8	28.1	85	121	98.8	100	100	100
36	209,731	2.5	5.6	15.3	30.7	86	84	99.2	100	100	100
37	208,831	2.8	6.2	16.8	33.5	87	71	100	100	100	100
38	203,212	3.1	6.9	18.5	36.4	88	39	100	100	100	100
39	193,878	3.5	7.6	20.3	39.5	89	25	100	100	100	100
40	184,428	3.9	8.4	22.3	42.8	90	21	100	100	100	100
41	175,324	4.3	9.3	24.5	46.2	91	11	100	100	100	100
42	167,077	4.8	10.3	26.8	49.8	92	10	100	100	100	100
43	161,468	5.3	11.4	29.3	53.4	93	11	100	100	100	100
44	156,512	5.9	12.6	32.0	57.2	94	6	100	100	100	100
45	153,606	6.5	13.9	34.8	61.0	95	1	100	100	100	100
46	150,835	7.2	15.3	37.8	64.8	96	2	100	100	100	100
47	147,314	8.0	16.9	41.0	68.6	97	2	100	100	100	100
48	142,807	8.8	18.6	44.4	72.3	98	-	100	100	100	100
49	136,957	9.8	20.4	47.8	76.0	99	2	100	100	100	100
50	130,776	10.8	22.4	51.4	79.4	100	1	100	100	100	100
