# Supplementary material for: Combining Charlson comorbidity and VACS indices improves prognostic accuracy for all-cause mortality for patients with and without HIV in the Veterans Health Administration
Source: Front Med (Lausanne). 2024 Jan 31;10:1342466. doi: 10.3389/fmed.2023.1342466 (PMC10864663; doi:10.3389/fmed.2023.1342466)

Supplementary Figure S1. Predicted mortality from development sample using VACS-CCI score overlaid with observed mortality from Kaplan-Meier estimates. Data points shown represent minimum of 10 deaths and 5

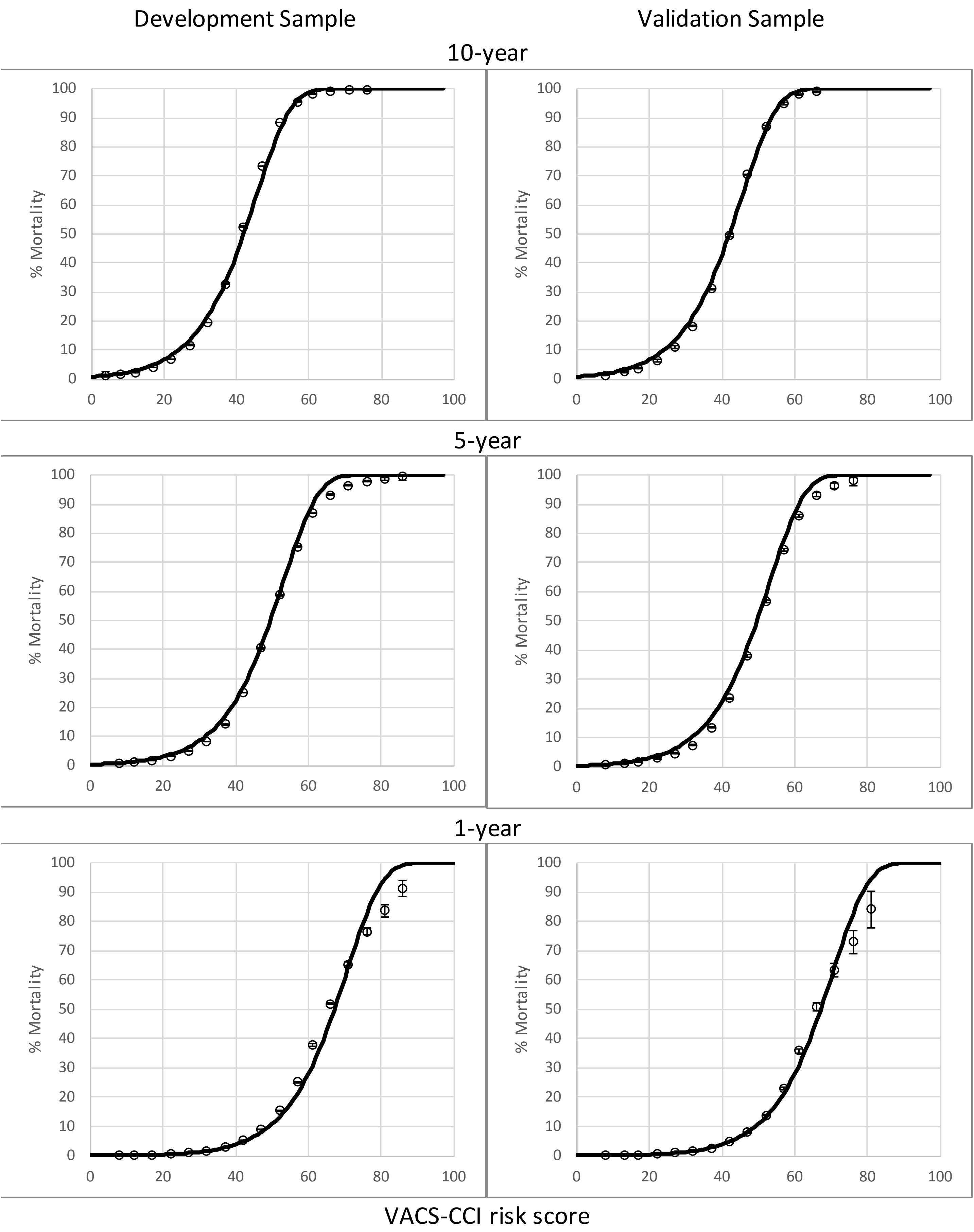

Supplement: Supplementary file 4 [file Image_1.pdf]
