# Supplementary figures and images for: Combining Charlson comorbidity and VACS indices improves prognostic accuracy for all-cause mortality for patients with and without HIV in the Veterans Health Administration
Source: Front Med (Lausanne). 2024 Jan 31;10:1342466. doi: 10.3389/fmed.2023.1342466 (PMC10864663; doi:10.3389/fmed.2023.1342466)

**Supplementary Figure 2. C-Statistics for Subgroups**

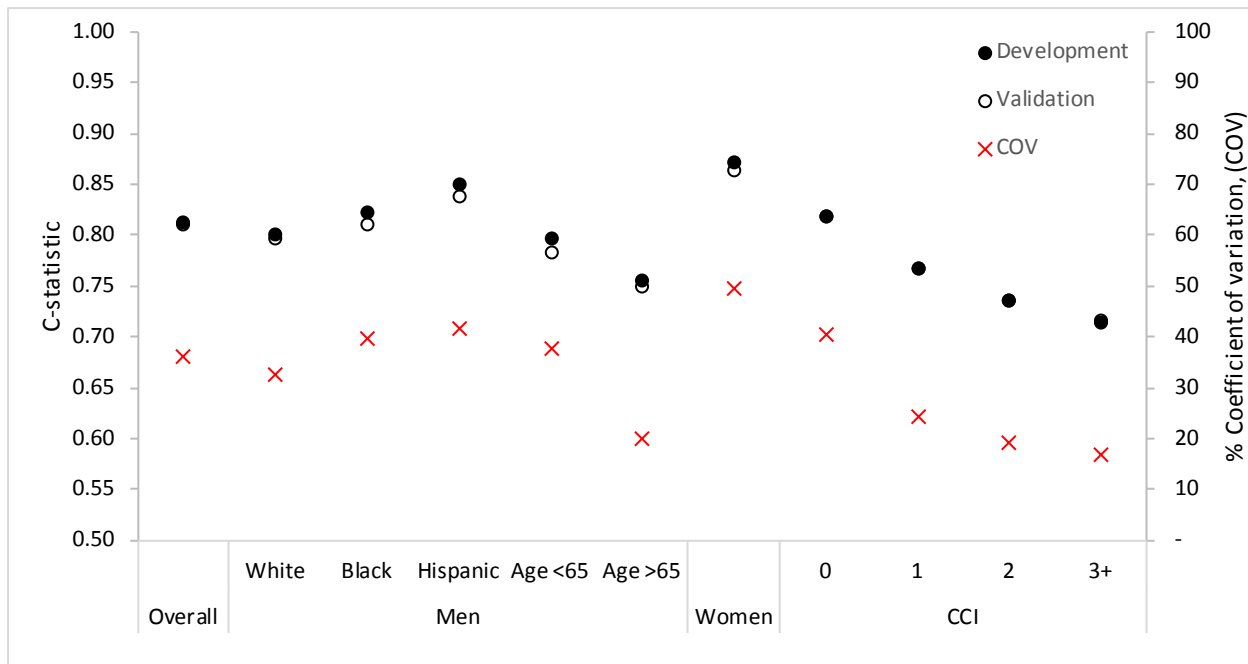

Supplement: Supplementary file 5 [file Image_2.pdf]
