# Supplementary material for: Combining Charlson comorbidity and VACS indices improves prognostic accuracy for all-cause mortality for patients with and without HIV in the Veterans Health Administration
Source: Front Med (Lausanne). 2024 Jan 31;10:1342466. doi: 10.3389/fmed.2023.1342466 (PMC10864663; doi:10.3389/fmed.2023.1342466)
Supplement: Supplementary file 2 [file Table_2.pdf]

Supplementary Table S2. ICD 9 and 10 codes used to define Charlson Comorbidity Index

ICD 9		ICD 10	
Code	Description	Code	Description
**Myocardial infarction (1 point)**			
410.x	Acute myocardial infarction	I21.x	ST elevation (stemi) myocardial infarction
412.	Old myocardial infarction	I21.A1	Myocardial infarction type 2
		I21.A9	Other myocardial infarction type
		I22.x	Subsequent st elevation (stemi) myocardial infarction of anterior wall
		I25.2	Old myocardial infarction
**Congestive heart failure (1 point)**			
398.91	Rheumatic heart failure (congestive)	I11.0	Hypertensive heart disease with heart failure
402.01	Hypertensive heart disease, malignant, with heart failure	I13.0	Hypertensive heart and chronic kidney disease with heart failure and stage 1 through stage 4 chronic
402.11	Hypertensive heart disease, benign, with heart failure	I13.2	Hypertensive heart and chronic kidney disease with heart failure and with stage 5 chronic kidney dis
402.91	Hypertensive heart disease, unspecified, with heart failure	I25.5	Ischemic cardiomyopathy
404.01	Hypertensive heart and renal disease, malignant, with heart failure	I42.0	Dilated cardiomyopathy
404.03	Hypertensive heart and renal disease, malignant, with heart failure and renal failure	I42.5	Other restrictive cardiomyopathy
404.11	Hypertensive heart and chronic kidney disease, benign, with heart failure and with chronic kidney di	I42.6	Alcoholic cardiomyopathy
404.13	Hypertensive heart and chronic kidney disease, benign, with heart failure and chronic kidney disease	I42.7	Cardiomyopathy due to drug and external agent
404.91	Hypertensive heart and chronic kidney disease, unspecified, with heart failure and with chronic kidn	I42.8	Other cardiomyopathies
404.93	Hypertensive heart and chronic kidney disease, unspecified, with heart failure and chronic kidney di	I42.9	Cardiomyopathy, unspecified
425.4	Other primary cardiomyopathies	I43.	Cardiomyopathy in diseases classified elsewhere
425.5	Alcoholic cardiomyopathy	I50.x	Heart failure
425.7	Nutritional and metabolic cardiomyopathy		
425.8	Cardiomyopathy in other diseases classified elsewhere		
425.9	Secondary cardiomyopathy, unspecified		
428.x	Heart failure		
**Peripheral vascular disease (PVD) (1 point)**			
437.3	Cerebral aneurysm, nonruptured	I70.x	Atherosclerosis (specified and unspecifed arteries, grafts)
440.x	Atherosclerosis (specified and unspecifed arteries, grafts)	I71.x	Arterial dissection, other and unspecified PVD
441.x	Aneurysm	I73.1	Thromboangiitis obliterans [buerger's disease]
443.x	Arterial dissection, other and unspecified PVD	I73.81	Erythromelalgia
447.1	Stricture of artery	I73.89	Other specified peripheral vascular diseases
557.1	Chronic vascular insufficiency of intestine	I73.9	Peripheral vascular disease, unspecified
557.9	Unspecified vascular insufficiency of intestine	I77.1	Stricture of artery
V43.4	Blood vessel replaced by other means	I79.0	Aneurysm of aorta in diseases classified elsewhere
		K55.1	Chronic vascular disorders of intestine
		K55.8	Other vascular disorders of intestine
		K55.9	Vascular disorder of intestine, unspecified
		Z95.8x	Presence of graft, defibrillator or heart assist device
		Z95.9	Presence of cardiac and vascular implant and graft, unspecified
**Cerebrovascular disease (1 point)**			
362.34	Transient retinal arterial occlusion	G45.x	Transient cerebral ischemic attack and related syndromes
430.x	Subarachnoid hemorrhage	G46.x	Other and specified vascular syndromes of brain in cerebrovascular diseases
431.x	Intracerebral hemorrhage	H34.0x - H	Retinal artery occlusions
432.x	Nontraumatic extradural hemorrhage	I60.x	Nontraumatic subarachnoid hemorrhage
433.x	Occlusion and stenosis of carotid or other artery	I61.x	Nontraumatic intracerebral hemorrhage
434.x	Cerebral thrombosis or embolism	I62.x	Other and unspecified nontraumatic intracranial hemorrhage
435.x	Transient cerebral ischemia	I63.x	Cerebral infarction
436.	Acute, but ill-defined, cerebrovascular disease	I65.x	Occlusion and stenosis of precerebral arteries, not resulting in cerebral infarction
437.x	Cerebral atherosclerosis and other cerebrovascular disease	I66.X	Occlusion and stenosis of cerebral arteries, not resulting in cerebral infarction
438.x	Late effects of cerebrovascular disease	I67.x	Other cerebrovascular diseases
		I68.x	Cerebrovascular disorders in diseases classified elsewhere
		I69.x	Sequelae of cerebrovascular disease
**Dementia (1 point)**			
290.x	Dementias	F01.5x	Vascular dementia
294.0	Amnestic syndrome	F02.8x	Dementia in other diseases classified elsewhere
294.1x	Dementia in conditions classified elsewhere	F03.9x	Unspecified dementia
294.2x	Dementia, unspecified.	G13.2	Systemic atrophy primarily affecting the central nervous system in myxedema
331.0	Alzheimer's disease	G13.8	Systemic atrophy primarily affecting central nervous system in other diseases classified elsewhere
331.1	Pick's disease	G30.x	Alzheimer's disease
331.2	Senile degeneration of brain	G31.0x	Pick's disease and other frontotemporal dementia
331.7	Cerebral degeneration in diseases classified elsewhere	G31.1	Senile degeneration of brain, not elsewhere classified
797.	Senility without mention of psychosis	G31.2	Degeneration of nervous system due to alcohol
		G31.83	Dementia with lewy bodies
**Chronic pulmonary disease (1 point)**			
416.8	Other chronic pulmonary heart diseases	I27.8x	Pulmonary heart diseases
416.9	Chronic pulmonary heart disease, unspecified	I27.9	Pulmonary heart disease, unspecified
490.x	Bronchitis, not specified as acute or chronic	J40.	Bronchitis, not specified as acute or chronic
491.x	Chronic bronchitis	J41.x	Chronic bronchitis
492.x	Emphysema	J42.x	Unspecified chronic bronchitis
493.x	Asthma	J43.x	Emphysema
494.x	Bronchiectasis	J44.x	Chronic obstructive pulmonary disease
495.x	Allergic alveolitis and pneumonitis	J45.x	Asthma
496.x	Chronic airway obstruction, not elsewhere classified	J47x	Bronchiectasis
500.x	Coal workers' pneumoconiosis	J60.	Coalworker's pneumoconiosis
501.x	Asbestosis	J61.	Pneumoconiosis due to asbestos and other mineral fibers
502.x	Pneumoconiosis due to other silica or silicates	J62.x	Pneumoconiosis due to dust containing silica
503.x	Pneumoconiosis due to other inorganic dust	J63.x	Pneumoconiosis due to inorganic dusts
504.x	Pneumonopathy due to inhalation of other dust	J64.	Unspecified pneumoconiosis
505.x	Pneumoconiosis, unspecified	J65.	Pneumoconiosis associated with tuberculosis
506.4	Chronic respiratory conditions due to fumes and vapors	J66.x	Airway disease due to organic dusts
508.1	Chronic and other pulmonary manifestations due to radiation	J67.x	Hypersensitivity pneumonitis due to organic dust
508.8	Respiratory conditions due to other specified external agents	J68.4	Chronic respiratory conditions due to chemicals, gases, fumes and vapors
		J70.1	Chronic and other pulmonary manifestations due to radiation
		J70.3	Chronic drug-induced interstitial lung disorders
**Rheumatic disease (1 point)**			
446.5	Giant cell arteritis	M05.0x	Felty's syndrome
710.0	Systemic lupus erythematosus	M05.1x	Rheumatoid lung disease
710.1	Systemic sclerosis	M05.2x	Rheumatoid vasculitis with rheumatoid arthritis
710.2	Sicca syndrome	M05.3x	Rheumatoid heart disease with rheumatoid arthritis
710.3	Dermatomyositis	M05.4x	Rheumatoid myopathy with rheumatoid arthritis
710.4	Polymyositis	M05.5x	Rheumatoid polyneuropathy with rheumatoid arthritis
714.0	Rheumatoid arthritis	M05.6x	Rheumatoid arthritis with involvement of other organs and systems
714.1	Felty's syndrome	M05.7x	Rheumatoid arthritis with rheumatoid factor without organ or systems involvement
714.2	Other rheumatoid arthritis with visceral or systemic involvement	M05.8x	Other rheumatoid arthritis with rheumatoid factor
714.81	Rheumatoid lung	M05.9x	Rheumatoid arthritis with rheumatoid factor, unspecified
714.89	Other specified inflammatory polyarthropathies	M06.0x	Rheumatoid arthritis without rheumatoid factor
725.	Polymyalgia rheumatica	M06.1	Adult-onset still's disease
		M06.2x	Rheumatoid bursitis
		M06.3x	Rheumatoid nodule
		M06.4	Inflammatory polyarthropathy
		M06.8x	Other specified rheumatoid arthritis
		M06.9	Rheumatoid arthritis, unspecified
		M31.5	Giant cell arteritis with polymyalgia rheumatica
		M32.x	Systemic lupus erythematosus
		M33.0x	Juvenile dermatopolymyositis
		M33.1x	Other dermatopolymyositis
		M33.2x	Polymyositis
		M33.9x	Dermatopolymyositis
		M34.x	Systemic sclerosis
		M35.x	Sicca syndrome/Sjögren syndrome, other systemic involvement of connective tissue
		M36.0	Dermato(poly)myositis in neoplastic disease
**Peptic ulcer disease (1 point)**			
531.x	Gastric ulcer	K25.x	Gastric ulcer
532.x	Duodenal ulcer	K26.x	Duodenal ulcer
533.x	Peptic ulcer	K27.x	Peptic ulcer
534.x	Gastrojejunal ulcer	K28.x	Gastrojejunal ulcer
**Diabetes without chronic complications (1 point)**			
250.0x	Diabetes mellitus without mention of complication	E10.1x	Type 1 diabetes mellitus with ketoacidosis
250.1x	Diabetes with ketoacidosis	E10.61x	Type 1 diabetes mellitus with diabetic arthropathy
250.2x	Diabetes with hyperosmolarity	E10.62x	Type 1 diabetes mellitus with skin complications
250.3x	Diabetes with other coma	E10.63x	Type 1 diabetes mellitus with oral complications
250.8x	Diabetes with other specified manifestations	E10.64x	Type 1 diabetes mellitus with hypoglycemia
250.9x	Diabetes with unspecified complication	E10.65	Type 1 diabetes mellitus with hyperglycemia
		E10.69	Type 1 diabetes mellitus with other specified complication
		E10.8	Type 1 diabetes mellitus with unspecified complications
		E10.9	Type 1 diabetes mellitus without complications
		E11.0x	Type 2 diabetes mellitus with hyperosmolarity
		E11.1x	Type 2 diabetes mellitus with ketoacidosis
		E11.61x	Type 2 diabetes mellitus with diabetic arthropathy
		E11.62x	Type 2 diabetes mellitus with skin complications
		E11.63x	Type 2 diabetes mellitus with oral complications
		E11.64x	Type 2 diabetes mellitus with hypoglycemia
		E11.65	Type 2 diabetes mellitus with hyperglycemia
		E11.69	Type 2 diabetes mellitus with other specified complication
		E11.8	Type 2 diabetes mellitus with unspecified complications
		E11.9	Type 2 diabetes mellitus without complications
		E13.0x	Other specified diabetes mellitus with hyperosmolarity
		E13.1x	Other specified diabetes mellitus with ketoacidosis
		E13.61x	Other specified diabetes mellitus with diabetic arthropathy
		E13.62x	Other specified diabetes mellitus with skin complications
		E13.63x	Other specified diabetes mellitus with oral complications
		E13.64x	Other specified diabetes mellitus with hypoglycemia
		E13.65	Other specified diabetes mellitus with hyperglycemia
		E13.69	Other specified diabetes mellitus with other specified complication
		E13.8	Other specified diabetes mellitus with unspecified complications
		E13.9	Other specified diabetes mellitus without complications
**Diabetes with chronic complications (2 points)**			
250.4x	Diabetes with renal manifestations	E10.21	Type 1 diabetes mellitus with diabetic nephropathy
250.5x	Diabetes with ophthalmic manifestations	E10.22	Type 1 diabetes mellitus with diabetic chronic kidney disease
250.6x	Diabetes with neurological manifestations	E10.29	Type 1 diabetes mellitus with other diabetic kidney complication
250.7x	Diabetes with peripheral circulatory disorders	E10.3x	Type 1 diabetes mellitus with diabetic retinopathy
		E10.4x	Type 1 diabetes mellitus with diabetic neuropathy
		E10.5x	Type 1 diabetes mellitus with diabetic peripheral angiopathy
		E11.21	Type 2 diabetes mellitus with diabetic nephropathy
		E11.22	Type 2 diabetes mellitus with diabetic chronic kidney disease
		E11.29	Type 2 diabetes mellitus with other diabetic kidney complication
		E11.3x	Type 2 diabetes mellitus with diabetic retinopathy
		E11.4x	Type 2 diabetes mellitus with diabetic neuropathy
		E11.5x	Type 2 diabetes mellitus with diabetic peripheral angiopathy
		E13.21	Other specified diabetes mellitus with diabetic nephropathy
		E13.22	Other specified diabetes mellitus with diabetic chronic kidney disease
		E13.29	Other specified diabetes mellitus with other diabetic kidney complication
		E13.3x	Other specified diabetes mellitus with diabetic retinopathy
		E13.4x	Other specified diabetes mellitus with diabetic neuropathy
		E13.5x	Other specified diabetes mellitus with diabetic peripheral angiopathy
**Hemiplegia or paraplegia (2 points)**			
334.1	Hereditary spastic paraplegia	G11.4	Hereditary spastic paraplegia
342.0x	Flaccid hemiplegia	G80.1	Spastic diplegic cerebral palsy
342.1x	Spastic hemiplegia	G80.2	Spastic hemiplegic cerebral palsy
342.8x	Other specified hemiplegia & hemiparesis	G81.x	Flaccid hemiplegia
342.9x	Hemiplegia, unspecified	G81.1x	Spastic hemiplegia
343.x	Congenital plegia	G81.9x	Hemiplegia, unspecified
344.0x	Quadriplegia	G82.2x	Paraplegia
344.1	Paraplegia	G82.5x	Quadriplegia
344.2	Diplegia of upper limbs	G83.0	Diplegia of upper limbs
344.3x	Monoplegia of lower limb	G83.1x	Monoplegia of lower limb
344.4x	Monoplegia of upper limb	G83.2x	Monoplegia of upper limb
344.6x	Cauda equina syndrome	G83.3x	Monoplegia, unspecified
344.9	Paralysis, unspecified	G83.4	Cauda equina syndrome
		G83.9	Paralytic syndrome, unspecified
**Renal disease, mild to moderate (1 point)**			
403.00	Hypertensive renal disease, malignant, without mention of renal failure	I12.9	Hypertensive chronic kidney disease with stage 1 through stage 4 chronic kidney disease, or unspecified
403.10	Hypertensive kidney disease, benign, without chronic kidney disease	I13.0	Hypertensive heart and chronic kidney disease with heart failure and stage 1 through stage 4 chronic
403.90	Hypertensive renal disease, unspecified, without mention of renal failure	I13.10	Hypertensive heart and chronic kidney disease without heart failure, with stage 1 through stage 4 ch
404.00	Hypertensive heart and renal disease, malignant, without mention of heart failure or renal failure	N03.x	Chronic nephritic syndrome
404.01	Hypertensive heart and renal disease, malignant, with heart failure	N05.x	Unspecified nephritic syndrome
404.10	Hypertensive heart and kidney disease, benign, without heart failure or chronic kidney disease	N18.1	Chronic kidney disease, stage 1
404.11	Hypertensive heart and chronic kidney disease, benign, with heart failure and with chronic kidney di	N18.2	Chronic kidney disease, stage 2 (mild)
404.90	Hypertensive heart and renal disease, unspecified, without mention of heart failure or renal failure	N18.3	Chronic kidney disease, stage 3 (moderate)
404.91	Hypertensive heart and chronic kidney disease, unspecified, with heart failure and with chronic kidn	N18.30	Chronic kidney disease, stage 3 unspecified
582.x	Chronic glomerulonephritis	N18.31	Chronic kidney disease, stage 3a
583.x	Nephritis and nephropathy, not specified as acute or chronic	N18.32	Chronic kidney disease, stage 3b
585.	Chronic renal failure	N18.4	Chronic kidney disease, stage 4 (severe)
585.1	Chronic kidney disease, stage i	N18.9	Chronic kidney disease, unspecified
585.2	Chronic kidney disease, stage ii (mild)	Z94.0	Kidney transplant status
585.3	Chronic kidney disease, stage iii (moderate)		
585.4	Chronic kidney disease, stage iv (severe)		
585.9	Chronic kidney disease, unspecified		
V42.0	Kidney replaced by transplant		
**Renal disease,severe (3 points)**			
403.01	Hypertensive chronic kidney disease, malignant, with chronic kidney disease stage v or end stage ren	I12.0	Hypertensive chronic kidney disease with stage 5 chronic kidney disease or end stage renal disease
403.11	Hypertensive chronic kidney disease, benign, with chronic kidney disease stage v or end stage renal	I13.11	Hypertensive heart and chronic kidney disease without heart failure, with stage 5 chronic kidney dis
403.91	Hypertensive renal disease, unspecified, with renal failure	I13.2	Hypertensive heart and chronic kidney disease with heart failure and with stage 5 chronic kidney dis
404.02	Hypertensive heart and kidney disease, malignant, with chronic kidney disease	N18.5	Chronic kidney disease, stage 5
404.03	Hypertensive heart and renal disease, malignant, with heart failure and renal failure	N18.6	End stage renal disease
404.12	Hypertensive heart and renal disease, benign, with renal failure	N19.	Unspecified kidney failure
404.13	Hypertensive heart and chronic kidney disease, benign, with heart failure and chronic kidney disease	N25.0	Renal osteodystrophy
404.92	Hypertensive heart and kidneydisease, unspecified, with chronic kidney disease	Z49.01	Encounter for fitting and adjustment of extracorporeal dialysis catheter
404.93	Hypertensive heart and chronic kidney disease, unspecified, with heart failure and chronic kidney di	Z49.02	Encounter for fitting and adjustment of peritoneal dialysis catheter
585.5	Chronic kidney disease, stage v	Z49.31	Encounter for adequacy testing for hemodialysis
585.6	End stage renal disease	Z49.32	Encounter for adequacy testing for peritoneal dialysis
586.	Renal failure, unspecified	Z99.2	Dependence on renal dialysis
588.0	Renal osteodystrophy		
V45.11	Renal dialysis status		
V45.12	Noncompliance with renal dialysis		
V56.0	Aftercare involving extracorporeal dialysis		
V56.2	Fitting and adjustment of peritoneal dialysis catheter		
V56.31	Encounter for adequacy testing for hemodialysis		
V56.32	Encounter for adequacy testing for peritoneal dialysis		
V56.8	Aftercare involving other dialysis		
**Liver disease, mild (1 point)**			
070.22	Chronic viral hepatitis B w/ hepatic coma w/o mention of hepatitis delta	B18.0	Chronic viral hepatitis B with delta-agent
070.23	Chronic viral hepatitis B w/ hepatic coma w/ hepatitis delta	B18.1	Chronic viral hepatitis B without delta-agent
070.32	Chronic viral hepatitis B w/o mention of hepatic coma w/o mention of hepatitis delta	B18.2	Chronic viral hepatitis C
070.33	Chronic viral hepatitis B w/o mention of hepatic coma w/ hepatitis delta	B18.8	Other chronic viral hepatitis
070.44	Chronic hepatitis C w/ hepatic coma	B18.9	Chronic viral hepatitis, unspecified
070.54	Chronic hepatitis C w/o mention of hepatic coma	K70.0	Alcoholic fatty liver
070.6	Unspecified viral hepatitis with hepatic coma	K70.1x	Alcoholic hepatitis
070.9	Unspecified viral hepatitis without mention of hepatic coma	K70.2	Alcoholic fibrosis and sclerosis of liver
570.	Acute and subacute necrosis of liver	K70.3x	Alcoholic cirrhosis of liver
571.0	Alcoholic fatty liver	K70.9	Alcoholic liver disease, unspecified
571.1	Acute alcoholic hepatitis	K71.3	Toxic liver disease with chronic persistent hepatitis
571.2	Alcoholic cirrhosis of liver	K71.4	Toxic liver disease with chronic lobular hepatitis
571.3	Alcoholic liver damage, unspecified	K71.5x	Toxic liver disease with chronic active hepatitis
571.40	Chronic hepatitis, unspecified	K71.7	Toxic liver disease with fibrosis and cirrhosis of liver
571.41	Chronic persistent hepatitis	K73.x	Chronic hepatitis, not elsewhere classified
571.42	Autoimmune hepatitis	K74.0x	Hepatic fibrosis
571.49	Other chronic hepatitis	K74.1	Hepatic sclerosis
571.5	Cirrhosis of liver without mention of alcohol	K74.2	Hepatic fibrosis with hepatic sclerosis
571.6	Biliary cirrhosis	K74.3	Primary biliary cirrhosis
571.8	Other chronic nonalcoholic liver disease	K74.4	Secondary biliary cirrhosis
571.9	Unspecified chronic liver disease without mention of alcohol	K74.5	Biliary cirrhosis, unspecified
573.3	Hepatitis, unspecified	K74.60	Unspecified cirrhosis of liver
573.4	Hepatic infarction	K74.69	Other cirrhosis of liver
573.8	Other specified disorders of liver	K75.4	Autoimmune hepatitis
573.9	Unspecified disorder of liver	K75.81	Nonalcoholic steatohepatitis (nash)
V42.7	Liver replaced by transplant	K76.0	Fatty (change of) liver, not elsewhere classified
		K76.2	Central hemorrhagic necrosis of liver
		K76.3	Infarction of liver
		K76.4	Peliosis hepatis
		K76.81	Hepatopulmonary syndrome
		K76.89	Other specified diseases of liver
		K76.9	Liver disease, unspecified
		Z94.4	Liver transplant status
**Liver disease, moderate to severe (3 points)**			
456.0	Esophageal varices with bleeding	I85.00	Esophageal varices without bleeding
456.1	Esophageal varices without mention of bleeding	I85.01	Esophageal varices with bleeding
456.20	Esophageal varices in diseases classified elsewhere, with bleeding	I86.4	Gastric varices
456.21	Esophageal varices in diseases classified elsewhere, without mention of bleeding	K70.4x	Alcoholic hepatic failure
572.2	Hepatic coma	K71.1x	Toxic liver disease with hepatic necrosis
572.3	Portal hypertension	K72.1x	Chronic hepatic failure
572.4	Hepatorenal syndrome	K72.9x	Hepatic failure, unspecified
572.8	Other sequelae of chronic liver disease	K76.5	Hepatic veno-occlusive disease
		K76.6	Portal hypertension
		K76.7	Hepatorenal syndrome
**Any malignancy (2 points)**			
140.x	Malignant neoplasm of lip	C00.x	Malignant neoplasm of lip
141.x	Malignant neoplasm of tongue	C01.	Malignant neoplasm of base of tongue
142.x	Malignant neoplasm of salivary gland	C02.x	Malignant neoplasm of tongue,
143.x	Malignant neoplasm of gum	C03.x	Malignant neoplasm of gum
144.x	Malignant neoplasm of floor of mouth	C04.x	Malignant neoplasm of floor of mouth
145.x	Malignant neoplasm of mouth	C05.x	Malignant neoplasm of palate
146.x	Malignant neoplasm of oropharynx	C06.x	Malignant neoplasm of mouth
147.x	Malignant neoplasm of nasopharynx	C07.	Malignant neoplasm of parotid gland
148.x	Malignant neoplasm of hypopharynx	C08.x	Malignant neoplasm of major salivary gland
149.x	Malignant neoplasm of other and ill-defined sites within the lip, oral cavity, and pharynx	C09.x	Malignant neoplasm of tonsil
150.x	Malignant neoplasm of esophagus,	C10.x	Malignant neoplasm of oropharynx
151.x	Malignant neoplasm of stomach	C11.x	Malignant neoplasm of nasopharynx
152.x	Malignant neoplasm of small intestine	C12.	Malignant neoplasm of pyriform sinus
153.x	Malignant neoplasm of colon	C13.x	Malignant neoplasm of hypopharynx
154.x	Malignant neoplasm of rectum, rectosigmoid junction, and anus	C14.x	Malignant neoplasm of pharynx
155.0	Malignant neoplasm of liver, primary	C15.x	Malignant neoplasm of esophagus
155.1	Malignant neoplasm of intrahepatic bile ducts	C16.x	Malignant neoplasm of stomach
155.2	Malignant neoplasm of liver, not specified as primary or secondary	C17.x	Malignant neoplasm of small intestine
156.x	Malignant neoplasm of gallbladder and extrahepatic bile ducts	C18.x	Malignant neoplasm of colon
157.x	Malignant neoplasm of pancreas	C19.	Malignant neoplasm of rectosigmoid junction
158.x	Malignant neoplasm of peritoneum or retroperitoneum	C20.	Malignant neoplasm of rectum
159.x	Malignant neoplasm ofother and ill-defined sites within the digestive organs organs and peritoneum	C21.x	Malignant neoplasm of rectum, anus and anal canal
160.x	Malignant neoplasm of nasal cavities middle ear and accessory sinuses	C22.x	Malignant neoplasm of liver
161.x	Malignant neoplasm of larynx	C23.	Malignant neoplasm of gallbladder
162.x	Malignant neoplasm of bronchus and lung	C24.x	Malignant neoplasm of biliary tract
163.x	Malignant neoplasm of pleura	C25.x	Malignant neoplasm of pancreas
164.x	Malignant neoplasm of thymus heart and mediastinum	C26.0	Malignant neoplasm of intestinal tract, part unspecified
165x	Malignant neoplasm of other and ill-defined sites within the respiratory system	C26.1	Malignant neoplasm of spleen
170.x	Malignant neoplasm of bone and articular cartilage	C26.9	Malignant neoplasm of ill-defined sites within the digestive system
171.x	Malignant neoplasm of connective and other soft tissue	C30.x	Malignant neoplasm of nasal cavity or middle ear
172.x	Malignant melanoma	C31.x	Malignant neoplasm of accessory sinus
174.x	Malignant neoplasm of female breast	C32.x	Malignant neoplasm of larynx
175.x	Malignant neoplasm of male breast	C33.	Malignant neoplasm of trachea
176.x	Kaposi's sarcoma	C34.0x	Malignant neoplasm of main bronchus
179.x	Malignant neoplasm of uterus	C34.1x	Malignant neoplasm of upper lobe, bronchus or lung
180.x	Malignant neoplasm of cervix uteri	C34.2	Malignant neoplasm of middle lobe, bronchus or lung
181.	Malignant neoplasm of placenta	C34.3x	Malignant neoplasm of lower lobe, bronchus or lung
182.x	Malignant neoplasm of corpus uteri	C34.8x	Malignant neoplasm of overlapping sites of bronchus and lung
183.x	Malignant neoplasm of ovary, fallopian tube uterine ligaments, uterine adnexa	C34.9x	Malignant neoplasm of unspecified part of bronchus or lung
184.x	Malignant neoplasm of female genital organ	C37.	Malignant neoplasm of thymus
185.	Malignant neoplasm of prostate	C38.x	Malignant neoplasm of heart, mediastinum and pleura
186.x	Malignant neoplasm of testis	C39.x	Malignant neoplasm of respiratory tract, part unspecified
187.x	Malignant neoplasm of male genital organ	C40.x	Malignant neoplasm of bones and articular cartilage
188.x	Malignant neoplasm of bladder	C41.x	Malignant neoplasm of other bone and articular cartilage
189.x	Malignant neoplasm of kidney or other urinary organ	C43.x	Malignant melanoma
190.x	Malignant neoplasm of eye	C45.x	Mesothelioma
191.x	Malignant neoplasm of brain	C46.x	Kaposi's sarcoma
192.x	Malignant neoplasm of nervous system other and unspecified	C47.x	Malignant neoplasm of peripheral nerves and autonomic nervous system
193.x	Malignant neoplasm of thyroid gland	C48.x	Malignant neoplasm of retroperitoneum and peritoneum
194.x	Malignant neoplasm of endocrine gland	C49.x	Malignant neoplasm of connective and soft tissue
195.x	Malignant neoplasm of other specified sites	C49.Ax	Gastrointestinal stromal tumor
199.1	Other malignant neoplasm of unspecified site	C50.x1x	Malignant neoplasm of female breast
200.0x	Reticulosarcoma	C50.x2x	Malignant neoplasm of male breast
200.1x	Lymphosarcoma	C51.x	Malignant neoplasm of vulva
200.2x	Burkitt's tumor or lymphoma	C52.	Malignant neoplasm of vagina
200.3x	Marginal zone lymphoma	C53.x	Malignant neoplasm of cervix uteri
200.4x	Mantle cell lymphoma	C54.x	Malignant neoplasm of corpus uteri
200.5x	Primary central nervous system lymphoma	C55.	Malignant neoplasm of uterus, part unspecified
200.6x	Anaplastic large cell lymphoma	C56.x	Malignant neoplasm of ovary
200.7x	Large cell lymphoma	C57.0x	Malignant neoplasm of fallopian tube
200.8x	Other named variants of lymphosarcoma and reticulosarcoma	C57.1x	Malignant neoplasm of broad ligament
201.0x	Hodgkin's paragranuloma	C57.2x	Malignant neoplasm of round ligament
201.1x	Hodgkin's granuloma	C57.3	Malignant neoplasm of parametrium
201.2x	Hodgkin's sarcoma	C57.4	Malignant neoplasm of uterine adnexa, unspecified
201.4x	Hodgkin's disease, lymphocytic-histiocytic predominance	C57.7	Malignant neoplasm of other specified female genital organs
201.5x	Hodgkin's disease, nodular sclerosis	C57.8	Malignant neoplasm of overlapping sites of female genital organs
201.6x	Hodgkin's disease, mixed cellularity	C57.9	Malignant neoplasm of female genital organ, unspecified
201.7x	Hodgkin's disease, lymphocytic depletion	C58.	Malignant neoplasm of placenta
201.9x	Hodgkin's disease, unspecified type	C60.x	Malignant neoplasm of penis
202.0x	Nodular lymphoma	C61.	Malignant neoplasm of prostate
202.1x	Mycosis fungoides	C62.x	Malignant neoplasm of testis
202.2x	Sezary's disease	C63.0x	Malignant neoplasm of epididymis
202.3x	Malignant histiocytosis	C63.1x	Malignant neoplasm of spermatic cord
202.4x	Leukemic reticuloendotheliosis	C63.2	Malignant neoplasm of scrotum
202.5x	Letterer-siwe disease	C63.7	Malignant neoplasm of other specified male genital organs
202.6x	Malignant mast cell tumors	C63.8	Malignant neoplasm of overlapping sites of male genital organs
202.7x	Peripheral t cell lymphoma	C63.9	Malignant neoplasm of male genital organ, unspecified
202.8x	Other malignant lymphomas	C64.1	Malignant neoplasm of kidney, except renal pelvis
202.9x	Other and unspecified malignant neoplasms of lymphoid and histiocytic tissue	C65.x	Malignant neoplasm of renal pelvis
203.0x	Multiple myeloma	C66.x	Malignant neoplasm of ureter
203.1x	Plasma cell leukemia	C67.x	Malignant neoplasm of bladder
203.8x	Other immunoproliferative neoplasms	C68.x	Malignant neoplasm of urinary organ, other and unspecified
204.0x	Acute lymphoid leukemia	C69.0x	Malignant neoplasm of conjunctiva
204.1x	Chronic lymphoid leukemia	C69.1x	Malignant neoplasm of cornea
204.2x	Subacute lymphoid leukemia	C69.2x	Malignant neoplasm of retina
204.8x	Other lymphoid leukemia	C69.3x	Malignant neoplasm of choroid
204.9x	Unspecified lymphoid leukemia	C69.4x	Malignant neoplasm of ciliary body
205.0x	Acute myeloid leukemia	C69.5x	Malignant neoplasm of lacrimal gland and duct
205.1x	Chronic myeloid leukemia	C69.6x	Malignant neoplasm of orbit
205.2x	Subacute myeloid leukemia	C69.8x	Malignant neoplasm of overlapping sites of eye and adnexa
205.3x	Myeloid sarcoma	C69.9x	Malignant neoplasm of unspecified site of eye
205.8x	Other myeloid leukemia	C70.x	Malignant neoplasm of meninges
205.9x	Unspecified myeloid leukemia	C71.x	Malignant neoplasm of brain
206.0x	Acute monocytic leukemia	C72.0	Malignant neoplasm of spinal cord
206.1x	Chronic monocytic leukemia	C72.1	Malignant neoplasm of cauda equina
206.2x	Subacute monocytic leukemia	C72.2x	Malignant neoplasm of olfactory nerve
206.8x	Other monocytic leukemia	C72.3x	Malignant neoplasm of optic nerve
206.9x	Unspecified monocytic leukemia	C72.4x	Malignant neoplasm of acoustic nerve
207.0x	Acute erythremia and erythroleukemia	C72.5x	Malignant neoplasm of other or unspecified cranial nerve
207.1x	Chronic erythremia	C72.9	Malignant neoplasm of central nervous system, unspecified
207.2x	Megakaryocytic leukemia	C73.	Malignant neoplasm of thyroid gland
207.8x	Other specified leukemia	C74.x	Malignant neoplasm adrenal gland
208.0x	Acute leukemia of unspecified cell type,	C75.x	Malignant neoplasm of endocrine gland
208.1x	Chronic leukemia of unspecified cell type	C76.x	Malignant neoplasm of other specified and ill-defined sites
208.2x	Subacute leukemia of unspecified cell type	C80.1	Malignant (primary) neoplasm, unspecified
208.8x	Other leukemia of unspecified cell type	C81.0x	Nodular lymphocyte predominant hodgkin lymphoma
208.9x	Unspecified leukemia	C81.1x	Nodular sclerosis classical hodgkin lymphoma
		C81.2x	Mixed cellularity classical hodgkin lymphoma
		C81.3x	Lymphocyte depleted classical hodgkin lymphoma
		C81.4x	Lymphocyte-rich classical hodgkin lymphoma
		C81.7x	Other classical hodgkin lymphoma
		C81.9x	Hodgkin lymphoma, unspecified
		C82.x	Follicular lymphoma
		C83.0x	Small cell B-cell lymphoma,
		C83.1x	Mantle cell lymphoma
		C83.3x	Diffuse large B-cell lymphoma, extranodal and solid organ sites
		C83.5x	Lymphoblastic (diffuse) lymphoma
		C83.7x	Burkitt lymphoma
		C83.8x	Other non-follicular lymphoma
		C83.9x	Non-follicular (diffuse) lymphoma
		C84.0x	Mycosis fungoides
		C84.1x	Sezary disease
		C84.4x	Peripheral T-cell lymphoma
		C84.6x	Anaplastic large cell lymphoma, alk-positive
		C84.7x	Anaplastic large cell lymphoma, alk-negative
		C84.9x	Mature T/NK-cell lymphomas, unspecified
		C84.Ax	Cutaneous T-cell lymphoma, unspecified
		C84.Zx	Other mature T/NK-cell lymphomas
		C85.1x	Unspecified B-cell lymphoma, unspecified site
		C85.2x	Mediastinal (thymic) large B-cell lymphoma
		C85.8x	Other specified types of non-hodgkin lymphoma
		C85.9x	Non-hodgkin lymphoma, unspecified
		C88.x	Malignant immunoproliferative disease
		C90.0x	Multiple myeloma
		C90.1x	Plasma cell leukemia
		C90.2x	Extramedullary plasmacytoma
		C90.3x	Solitary plasmacytoma
		C91.0x	Acute lymphoblastic leukemia
		C91.1x	Chronic lymphocytic leukemia of B-cell type
		C91.3x	Prolymphocytic leukemia of B-cell type
		C91.4x	Hairy cell leukemia
		C91.5x	Adult T-cell lymphoma/leukemia (HTLV-1-associated)
		C91.6x	Prolymphocytic leukemia of T-cell type
		C91.9x	Lymphoid leukemia, unspecified
		C91.Ax	Mature B-cell leukemia Burkitt-type
		C91.Zx	Other lymphoid leukemia
		C92.0x	Acute myeloblastic leukemia
		C92.1x	Chronic myeloid leukemia, BCR/ABL-positive
		C92.2x	Atypical chronic myeloid leukemia, BCR/ABL-negative
		C92.3x	Myeloid sarcoma
		C92.4x	Acute promyelocytic leukemia
		C92.5x	Acute myelomonocytic leukemia
		C92.6x	Acute myeloid leukemia with 11q23-abnormality
		C92.9x	Myeloid leukemia, unspecified
		C92.Ax	Acute myeloid leukemia with multilineage dysplasia
		C92.Zx	Other myeloid leukemia
		C93.0x	Acute monoblastic/monocytic leukemia
		C93.1x	Chronic myelomonocytic leukemia
		C93.3x	Juvenile myelomonocytic leukemia
		C93.9x	Monocytic leukemia, unspecified
		C93.Zx	Other monocytic leukemia
		C94.0x	Acute erythroid leukemia
		C94.2x	Acute megakaryoblastic leukemia
		C94.3x	Mast cell leukemia
		C94.4x	Acute panmyelosis with myelofibrosis
		C94.6	Myelodysplastic disease, not classified
		C94.8x	Other specified leukemias
		C95.0x	Acute leukemia of unspecified cell type
		C95.1x	Chronic leukemia of unspecified cell type
		C95.9x	Leukemia, unspecified
		C96.x	Malignant neoplasm of lymphoid, hematopoietic and related tissue
**Metastatic solid tumor (6 points)**			
196.x	Secondary and unspecified malignant neoplasm of lymph nodes	C77.x	Secondary and unspecified malignant neoplasm of lymph nodes
197.0	Secondary malignant neoplasm of lung	C78.0x	Secondary malignant neoplasm of lung
197.1	Secondary malignant neoplasm of mediastinum	C78.1	Secondary malignant neoplasm of mediastinum
197.2	Secondary malignant neoplasm of pleura	C78.2	Secondary malignant neoplasm of pleura
197.3	Secondary malignant neoplasm of other respiratory organs	C78.30	Secondary malignant neoplasm of unspecified respiratory organ
197.4	Secondary malignant neoplasm of small intestine including duodenum	C78.39	Secondary malignant neoplasm of other respiratory organs
197.5	Secondary malignant neoplasm of large intestine and rectum	C78.4	Secondary malignant neoplasm of small intestine
197.6	Secondary malignant neoplasm of retroperitoneum and peritoneum	C78.5	Secondary malignant neoplasm of large intestine and rectum
197.7	Malignant neoplasm of liver, specified as secondary	C78.6	Secondary malignant neoplasm of retroperitoneum and peritoneum
197.8	Secondary malignant neoplasm of other digestive organs organs and spleen	C78.7	Secondary malignant neoplasm of liver and intrahepatic bile duct
198.0	Secondary malignant neoplasm of kidney	C78.80	Secondary malignant neoplasm of unspecified digestive organ
198.1	Secondary malignant neoplasm of other urinary organs	C78.89	Secondary malignant neoplasm of other digestive organs
198.2	Secondary malignant neoplasm of skin	C79.0x	Secondary malignant neoplasm of kidney and renal pelvis
198.3	Secondary malignant neoplasm of brain and spinal cord	C79.10	Secondary malignant neoplasm of unspecified urinary organs
198.4	Secondary malignant neoplasm of other parts of nervous system	C79.11	Secondary malignant neoplasm of bladder
198.5	Secondary malignant neoplasm of bone and bone marrow	C79.19	Secondary malignant neoplasm of other urinary organs
198.6	Secondary malignant neoplasm of ovary	C79.2	Secondary malignant neoplasm of skin
198.7	Secondary malignant neoplasm of adrenal gland	C79.31	Secondary malignant neoplasm of brain
198.81	Secondary malignant neoplasm of breast	C79.32	Secondary malignant neoplasm of cerebral meninges
198.82	Secondary malignant neoplasm of genital organs	C79.40	Secondary malignant neoplasm of unspecified part of nervous system
198.89	Secondary malignant neoplasm of other specified sites	C79.49	Secondary malignant neoplasm of other parts of nervous system
199.0	Disseminated malignant neoplasm	C79.51	Secondary malignant neoplasm of bone
		C79.52	Secondary malignant neoplasm of bone marrow
		C79.6x	Secondary malignant neoplasm of ovary
		C79.7x	Secondary malignant neoplasm of adrenal gland
		C79.81	Secondary malignant neoplasm of breast
		C79.82	Secondary malignant neoplasm of genital organs
		C79.89	Secondary malignant neoplasm of other specified sites
		C79.9	Secondary malignant neoplasm of unspecified site
		C80.0	Disseminated malignant neoplasm, unspecified
**HIV (3 points)**			
042.x	Human immunodeficiency virus (HIV) disease	B20.	Human immunodeficiency virus [HIV] disease

Sources for ICD-9 and ICD-10 diagnosis codes

Quan H, Sundararajan V, Halfon P, Fong A, Burnand B, Luthi JC, Saunders LD, Beck CA, Feasby TE, Ghali WA. Coding algorithms for defining comorbidities in ICD-9-CM and ICD-10 administrative data. Med Care. 2005 Nov;43(11):1130-9.

Glasheen WP, Cordier T, Gumpina R, Haugh G, Davis J, Renda A. Charlson Comorbidity Index: ICD-9 Update and ICD-10 Translation. Am Health Drug Benefits. 2019 Jun-Jul;12(4):188-197.

Beyrer J, Manjelievskaia J, Bonafede M, Lenhart G, Nolot S, Haldane D, Johnston J. Validation of an International Classification of Disease, 10th revision coding adaptation for the Charlson Comorbidity Index in United States healthcare claims data. Pharmacoepidemiol Drug Saf. 2021 May;30(5):582-593.
